# Temporal dynamics of geothermal microbial communities in Aotearoa-New Zealand

**DOI:** 10.3389/fmicb.2023.1094311

**Published:** 2023-03-15

**Authors:** Jean F. Power, Caitlin L. Lowe, Carlo R. Carere, Ian R. McDonald, S. Craig Cary, Matthew B. Stott

**Affiliations:** ^1^Thermophile Research Unit, Te Aka Mātuatua | School of Science, Te Whare Wānanga o Waikato | University of Waikato, Hamilton, New Zealand; ^2^Te Tari Pūhanga Tukanga Matū | Department of Chemical and Process Engineering, Te Whare Wānanga o Waitaha | University of Canterbury, Christchurch, New Zealand; ^3^Biomolecular Interaction Centre, Te Whare Wānanga o Waitaha | University of Canterbury, Christchurch, Aotearoa-New Zealand; ^4^Te Kura Pūtaiao Koiora | School of Biological Sciences, Te Whare Wānanga o Waitaha | University of Canterbury, Christchurch, New Zealand

**Keywords:** geothermal microbiology, temporal biogeography, microbial biogeography, extremophiles, hot springs, Aotearoa-New Zealand

## Abstract

Microbial biogeography studies, in particular for geothermal-associated habitats, have focused on spatial patterns and/or individual sites, which have limited ability to describe the dynamics of ecosystem behaviour. Here, we report the first comprehensive temporal study of bacterial and archaeal communities from an extensive range of geothermal features in Aotearoa-New Zealand. One hundred and fifteen water column samples from 31 geothermal ecosystems were taken over a 34-month period to ascertain microbial community stability (control sites), community response to both natural and anthropogenic disturbances in the local environment (disturbed sites) and temporal variation in spring diversity across different pH values (pH 3, 5, 7, 9) all at a similar temperature of 60–70°C (pH sites). Identical methodologies were employed to measure microbial diversity *via* 16S rRNA gene amplicon sequencing, along with 44 physicochemical parameters from each feature, to ensure confidence in comparing samples across timeframes. Our results indicated temperature and associated groundwater physicochemistry were the most likely parameters to vary stochastically in these geothermal features, with community abundances rather than composition more readily affected by a changing environment. However, variation in pH (pH ±1) had a more significant effect on community structure than temperature (±20°C), with alpha diversity failing to adequately measure temporal microbial disparity in geothermal features outside of circumneutral conditions. While a substantial physicochemical disturbance was required to shift community structures at the phylum level, geothermal ecosystems were resilient at this broad taxonomic rank and returned to a pre-disturbed state if environmental conditions re-established. These findings highlight the diverse controls between different microbial communities within the same habitat-type, expanding our understanding of temporal dynamics in extreme ecosystems.

## Introduction

Microbial biogeography studies patterns and processes of diversity across space and time. While spatial studies focus on contemporary community structures and allow direct comparison between target ecosystems, this type of analysis only captures a snapshot of community diversity and fails to consider the dynamic nature of microbial assemblages (Ju and Zhang, 2014; Fuhrman et al., 2015). A true description of local diversity requires both spatial and temporal examination to obtain a complete picture of habitat structure (Lauber et al., 2013). Neutral processes, such as immigration/extinction of species and population drift, have been shown to effect community abundances over time (Lee et al., 2013; Nemergut et al., 2013). Physicochemical flux can also alter microbial communities and/or populations in an ecosystem (Nguyen et al., 2021), with some taxonomic groups acting as indicators for overall community change (Fortunato et al., 2013; Cram et al., 2015). Interestingly, sampling time can affect spatial difference between microbial communities as sufficient time is needed to build felicitous composition from migration (Winter et al., 2013; Louca, 2022), which reinforces the use of both spatial and temporal scales for microbial ecology studies (Ladau et al., 2013; Roeselers et al., 2015). This includes understanding the distinction between stochastic and deterministic change to obtain a more accurate representation of ecosystem behaviour.

Previous research into temporal dynamics in geothermal ecosystems, including microbial mats, sediments and water columns, has presented conflicting results on the processes driving microbial community variation. Little to no disparity in microbial community structure was observed in geothermal mats across multiple temporal scales from three separate studies in Yellowstone National Park (YNP; Ferris and Ward, 1997; Norris et al., 2002; Bowen De León et al., 2013). A 5-year study across the water column of Boiling Springs Lake in the USA again revealed a stable community despite a significant seasonal temperature cycle (Siering et al., 2013). Nineteen geothermal features in Croatia also demonstrated a lack of seasonality within resident microbial community structures (Mitrović et al., 2022). However, other studies have shown a seasonality effect in microbial mats, water and sediment samples from geothermal features, where seasons coincided with dichotomous weather patterns (e.g., monsoon rainfall or snowmelt; Lacap et al., 2007; Briggs et al., 2014; Colman et al., 2021). Time-dependent differences to alpha diversity (i.e., richness and relative abundances), but not community dissimilarity have been reported in two separate studies (Lacap et al., 2007; Wang et al., 2014). Geothermal eukaryotic algae (order Cyanidiales) have been shown to increase in relative abundance and photosynthetic activity with sunlight during summer (Lehr et al., 2007), while temperature induced community variation across seasons in microbial mats from the Patagonian Andes (Mackenzie et al., 2013). Wang et al. (2014) discussed limited temporal change across entire communities of geothermal springs in China but demonstrated certain populations were susceptible to changes in pH, temperature and dissolved organic carbon. Within Aotearoa-New Zealand, temporal studies of geothermal microbial communities are limited to just two features: Champagne Pool (estimated volume 50,000 m^3^), where no changes to community structure, pH or temperature were reported in the water column over a 2-year period (Hetzer et al., 2007), and Inferno Crater Lake (estimated volume 18,500–65,200 m^3^), where community turnover was associated to recurrent geological reservoir cycling evident by extreme changes in water level (~8 m) and temperature (~30–70°C; Ward et al., 2017). There are multiple plausible reasons for the disparity in these studies; variation in community structure not recorded due to low resolution of taxa, small sample sizes, insufficient temporal scales, environmental differentiation and/or the absence of control sites. In order to generate a consensus in the drivers of geothermal microbial variation over time, further studies are needed, preferably with consistent methodologies across multiple hot spring types, to develop a more holistic view of ecosystem dynamics.

The 1,000 Springs Project (https://1000Springs.org.nz; Power et al., 2018) is a study investigating microbial biogeography across the Taupō Volcanic Zone (TVZ), a geothermal region in the North Island of Aotearoa-New Zealand (Supplementary Figure S1; Wilson et al., 1995). From July 2013 to April 2015, 1,019 spring samples were collected from 18 geothermal fields and 974 individual features across the TVZ. An initial biogeography study, conducted on 925 of these springs, reported pH to be the most significant physicochemical parameter driving spatial community composition (Power et al., 2018). To help address the knowledge gap on temporal biogeography in TVZ geothermal features, and to determine whether the spatial study represented a simplistic characterisation of these microbial communities, 19 sites from the 925 springs spatially investigated were also temporally sampled over a 34-month period to ascertain both long-term microbial community stability (i.e., control sites; Supplementary Figure S2) and reaction to physicochemical disturbances in the local environment (i.e., disturbed sites; Supplementary Figure S2). In addition, we sampled 12 geothermal features displaying moderate temperatures (60–70°C) across a range of pH values (pH 3, 5, 7 or 9) every 2 months for a 1-year period to assess drivers of community structure across the pH scale (i.e., pH sites; Supplementary Figure S2). Our results indicate that while pH remains a significant constraining parameter of microbial diversity in geothermal features on a spatial scale, temperature and groundwater physicochemical conditions are more likely to vary stochastically with time; resulting in a dynamic system where abundance levels of resident populations respond to these changes in the local environment.

## Materials and methods

### Sample description and collection

All samples were collected from the water columns of geothermal features within the Taupō Volcanic Zone (TVZ), Aotearoa-New Zealand (Supplementary Figure S1), as per detailed methodologies outlined in Power et al. (2018). We gathered three different categories of temporal samples to define whether and how, geothermal microbial communities changed over time, and what the primary drivers of these changes, if any, could be (Supplementary Figure S2). The three categories were defined as follows: category A—natural microbial variation in geothermal features with both known and unknown physicochemical source fluid (control sites; comprising five geothermal features and 13 samples); category B—both natural and anthropogenic disturbances to geothermal features (disturbed sites; comprising 14 geothermal features and 39 samples); and category C – microbial variation over time in geothermal features with different pH values (pH sites; comprising 12 geothermal features and 69 samples). Category A and B samples were collected between July 2013 and October 2016, and have the sample prefix P1 (Supplementary Tables S1–S3). Category C samples were collected between December 2015 and October 2016, and have the prefix P2 (Supplementary Table S4).

Category A (control sites) represents 13 samples from five distinct geothermal features. Three of these sites (Wairakei Features 5, 13 and 14; Wairakei-Tauhara geothermal field) were sampled twice within a 5-month period on the basis of deeply-sourced hydrothermal fluid inputs presenting minimal interaction with groundwater (Colman et al., 2019). These features are sourced from the Wairakei steam field injection pipe network (Thain and Carey, 2009), where surface fluid originates from a depth of at least 1,500 m (Bignall et al., 2010). A fourth spring (Radiata Pool, Ngatamariki geothermal field) was sampled twice 10-month apart, with elevated concentrations of bicarbonate (519 ppm) suggesting a longer subsurface residence time for the water column than previous control sites (Reyes et al., 2010). The fifth control site, Champagne Pool from the Waiotapu geothermal field, was selected to investigate the influence of seasonality on microbial communities within a large (~65 m diameter, ~50,000 m^3^ volume), well-established (ca. 900 years ago) and deeply sourced geothermal spring (Hetzer et al., 2007; Gallagher et al., 2020). This feature was sampled five times within a 16-month period.

**Figure 1:**
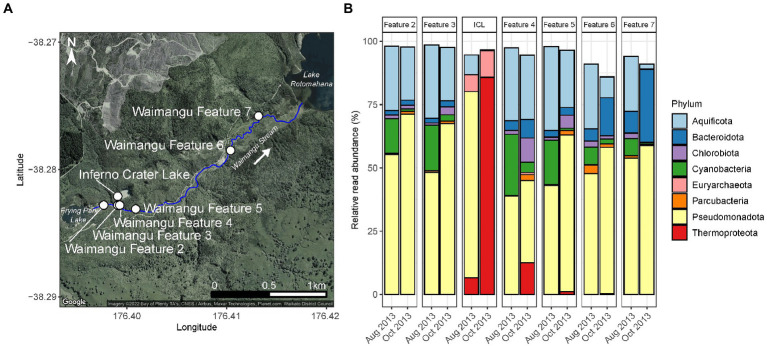
Short-term, natural disturbance of Waimangu Stream by Inferno Crater Lake. **(A)** Waimangu Stream (outlined by the solid blue line) originates from Frying Pan Lake and flows down a rift valley to Lake Rotomahana. Sampling locations along the stream (Waimangu Features 2–7) are depicted by white circles, along with Inferno Crater Lake which overflows into Waimangu Stream every ~30–40 days. Waimangu Features 2 and 3 are upstream of the Inferno Crater Lake overflow (by ~150 and 10 m, respectively), while Waimangu Features 4–7 are downstream (by ~35, 200, 1,400, and 1,950m, respectively). **(B)** Microbial community composition and relative abundance of Waimangu Stream features and Inferno Crater Lake (ICL) before (August 2013) and during (October 2013) the disturbance, measured by amplicon sequencing of the 16S rRNA gene. Only phyla >1% average relative abundance across all samples in this category are shown.

**Figure 2:**
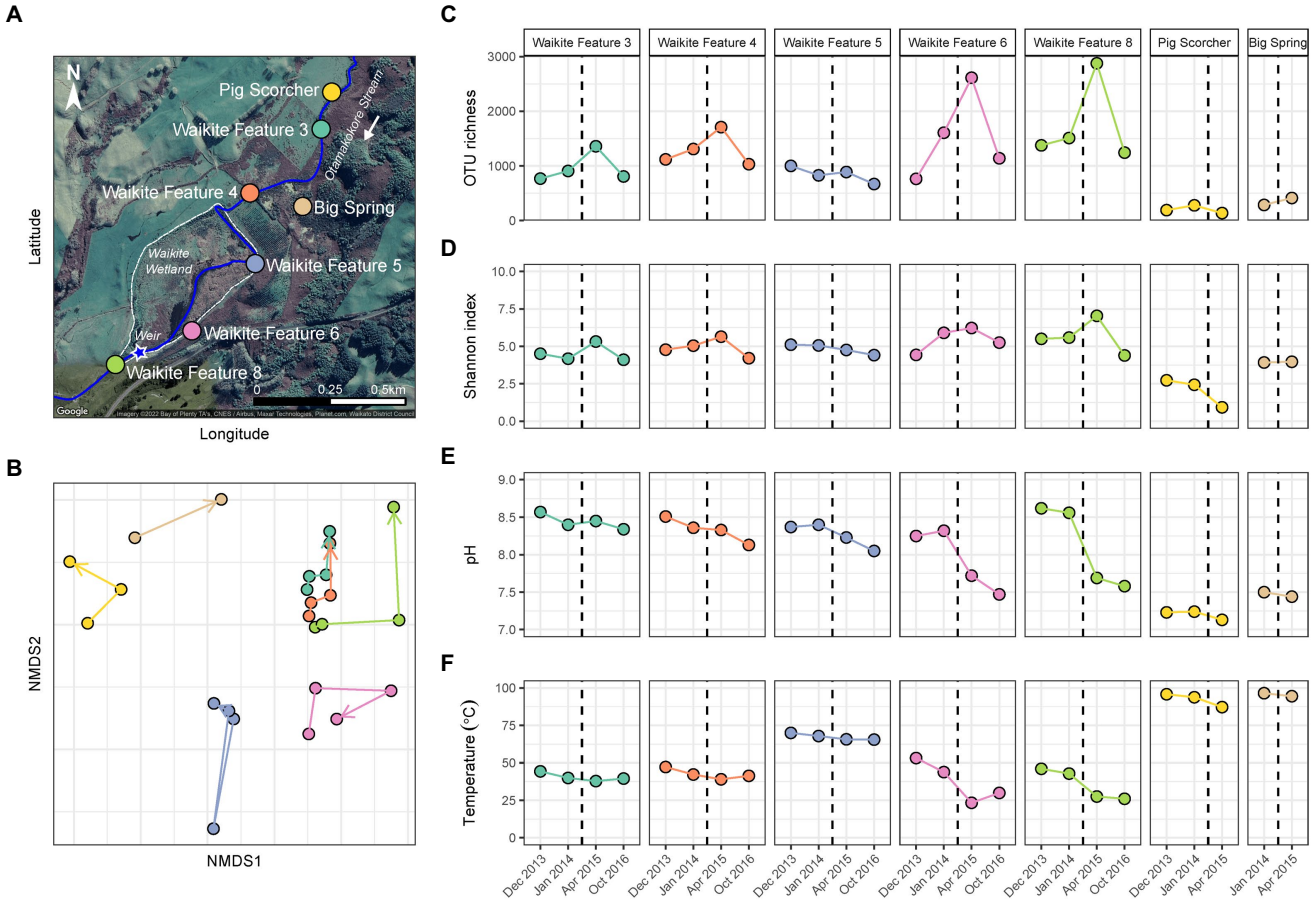
Temporal microbial diversity and physicochemistry of the Waikite wetland during restoration. **(A)** Aerial map of the Waikite geothermal field, including the wetland (dashed white line) and Otamakokore Stream (outlined by the solid blue line). The location of the weir is highlighted by the star, with stream sites and geothermal features sampled distinguished by colour. **(B)** Non-metric multidimensional scaling (NMDS; *n* = 25, stress = 0.11) of beta diversity, calculated using Bray-Curtis dissimilarities between 16S rRNA gene sequences of microbial communities in these features. Samples are coloured by feature name, with time indicated by arrows. **(C)** Variation in alpha diversity of samples measured *via* OTU richness (i.e., the number of OTUs per community). **(D)** Alpha diversity was also measured by the Shannon diversity index to indicate evenness of the communities over time. **(E)** The pH of features at the time of sampling. **(F)** Temperature variation of all sites during the wetland restoration. The black dashed line indicates the construction of a weir in the southwest corner of the wetland.

Category B samples (disturbed sites) were collected from two geothermal fields before and after a natural, short-term (i.e., pulse), or an anthropogenic, long-term (i.e., press) disturbance (Shade et al., 2012) to the resident microbial communities. The first set of sites were sampled from the Waimangu geothermal field (Figure 1), where Waimangu Stream (a.k.a. Hot Water Creek) is naturally disturbed every ~30–40 days by Inferno Crater Lake, a geothermal spring that cycles in both temperature (~30–70°C; Supplementary Figure S3) and water level (~8 m; Ward et al., 2017). The stream is continuously sourced (~104 l s^−1^) from Frying Pan Lake (~45°C, pH 7, and 200,000 m^3^ in volume), and experiences additional discontinuous sourcing (~79 l s^−1^) from Inferno Crater Lake (~70°C, pH 2 and 65,200 m^3^ in volume) for 1–2 days when the spring overflows at the end of a geological cycle (Scott, 1994). Six sites were sampled along the stream (two sites upstream of the Inferno Crater Lake overflow by 10 and 150 m, and four sites downstream of the overflow by 35, 200, 1,400, and 1,950 m), along with Inferno Crater Lake, both before (August 2013) and during overflow (October 2013), resulting in 14 samples for analysis. The second set of category B sites was collected from the Waikite geothermal field (Figure 2) which was undergoing a long-term wetland restoration (Reeves et al., 2018). Historically, the area was altered to create farming pasture by diverting Otamakokore Stream and draining the wetland, and rehabilitation to reverse these processes began in 2009. This included the installation of a weir on the stream in 2014, which raised the water level of the wetland by ~1.17 m (Reeves et al., 2018). Seven sites across the geothermal field were sampled twice before (December 2013 and January 2014), and twice after this disturbance (April 2015 and October 2016); one site upstream from the wetland (Waikite Feature 3), the inlet to the wetland (Waikite Feature 4), two sites within the wetland (Waikite Features 5 and 6), the outlet to the wetland (Waikite Feature 8) and two geothermal springs independently situated near the wetland, colloquially known as ‘Pig Scorcher’ and ‘Big Spring’ (Figure 2). These two sites were sampled to check if alterations to the wetland affected other features within the geothermal field, with reduced timepoints taken for health and safety reasons. We sampled both five- and 23-months post-weir installation, with a total of 25 samples from Waikite used for subsequent analysis.

Finally, category C samples (pH sites) targeted 12 geothermal features within the same temperature range (60–70°C) but at different pH levels (pH 3, 5, 7 and 9). Our spatial study on the microbial communities of 925 geothermal springs across the TVZ demonstrated pH as the major driver of diversity in these ecosystems (Power et al., 2018). Thus, to investigate whether pH had a similar effect on geothermal communities temporally, three features for each pH group were sampled from the Rotorua, Waiotapu, Atiamuri and Ohaaki geothermal fields (Supplementary Figure S1). To minimise the influence of temperature on community structure, features were selected between 60 and 70°C. This also allowed for decreased taxa numbers common to hot temperature springs to robustly identify microbial community change. The 12 sites were sampled every 2 months from December 2015 to October 2016, with three sites inaccessible in December 2015.

### Microbial and physicochemical analyses

Microbial DNA extraction, sequencing, processing and the measurement of 44 physicochemical parameters (listed below) from each sample were performed as described by Power et al. (2018), unless otherwise stated. Briefly, the 16S rRNA gene was analysed using the original Earth Microbiome Project primers (Thompson et al., 2017) and the Ion PGM system for next-generation sequencing. To remain consistent with the initial spatial biogeography study on these ecosystems (Power et al., 2018), raw sequences were processed using USEARCH (Edgar, 2013) and QIIME (Caporaso et al., 2010) to generate operational taxonomic units (OTUs) and respective read abundances, with taxonomy assigned using the RDP classifier (Wang et al., 2007) and the SILVA database v123 (Quast et al., 2013). Single rarefaction to the lowest sequence read count that did not compromise OTU diversity (*n* = 11,131 and 5,000 sequencing reads for categories A and B, and category C, respectively) was applied to all samples, with these sub-sampled reads used to calculate diversity measures. Conductivity (COND), oxidation–reduction potential (ORP) and pH were determined using a Hanna Instruments multiparameter field meter (Woonsocket, RI, United States), with spring temperature (TEMP) measured by a Fluke 51-II thermocouple (Everett, WA, United States). Inductively coupled plasma-mass spectrometry (ICP-MS), UV–vis spectrometry, titration and ion chromatography were used to measure the following aqueous metals and non-metals: aluminium (Al), ammonium (NH_4_^+^), arsenic (As), barium (Ba), bicarbonate (HCO_3_^−^), boron (B), bromine (Br), cadmium (Cd), caesium (Cs), calcium (Ca), chloride (Cl^−^), chromium (Cr), cobalt (Co), copper (Cu), ferrous iron (Fe^2+^), hydrogen sulphide (H_2_S), iron (Fe), lead (Pb), lithium (Li), magnesium (Mg), manganese (Mn), mercury (Hg), molybdate (Mo), nickel (Ni), nitrate (NO_3_^−^), nitrite (NO_2_^−^), phosphate (PO_4_^3−^), potassium (K), rubidium (Rb), selenium (Se), silicon (Si), silver (Ag), sodium (Na), strontium (Sr), sulphate (SO_4_^2−^), sulphur (S), thallium (Tl), uranium (U), vanadium (V), and zinc (Zn). In addition, six variables were analysed for category C samples. These included rainfall data (December 2015 to October 2016) obtained from the CliFlo National Climate Database [National Institute of Water and Atmospheric Research (NIWA), 2016]. Total daily depth (mm) was recorded from 00:00 to 23:59 New Zealand Standard Time (NZST) of each sampling date at the Whakarewarewa, Rotorua weather station (EK577135). The average daily water level depth (m) of five geothermal monitoring bores (M24-28) from the Rotorua geothermal field was also retrieved through the Environmental Data Portal managed by the Bay of Plenty Regional Council (BOPRC) (2016).

### Statistical analyses

All statistical analyses and figures were produced using R v4.0.3 (R Core Team, 2020) and the phyloseq package v1.32.0 (McMurdie and Holmes, 2013), unless otherwise stated. Operational taxonomic units (OTUs) generated from processing of the 16S rRNA gene sequences, and associated sample metadata, were imported into R using the import_biom function. The dataset was then divided into either category A, B or C using the subset_samples function. To assess variation in the read abundances of dominant taxa, OTUs in each sample were agglomerated to corresponding phylum (or genus) using the tax_glom function (with the argument NArm = FALSE), with reads transformed to percentage relative abundance using the transform_sample_counts function. Only microbial taxa with an average relative abundance of >1% were retained to allow robust identification of fluctuations to dominant community members (using the filter_taxa function), with plot_bar implemented to visualise these compositions over time. Using original OTUs (prior to phylum agglomeration), alpha diversity *via* OTU richness (i.e., number of OTUs) and evenness (i.e., Shannon diversity index) was calculated using the estimate_richness function. The package ggplot2 v3.3.2 (Wickham, 2016) was used to plot alpha diversity and physicochemical parameters (e.g., pH and temperature) over time. Beta diversity between spring communities was generated *via* Bray-Curtis dissimilarities using the vegdist function (with the “bray” method) from the vegan package v2.5–6 (Oksanen et al., 2019). Non-metric multidimensional scaling (NMDS) was implemented to visualise these dissimilarities *via* the metaMDS function (*k* = 2), also from the vegan package, in conjunction with ggplot2. Correlation testing between alpha diversity and spring physicochemistry was performed using the dist function with the “euclidean” dissimilarity index, and the cor.test function with Spearman’s correlation coefficient (ρ) from the base R stats package. For beta diversity, Bray-Curtis dissimilarities were checked for correlation with spring physicochemistry using the mantel function from vegan, again with Spearman’s correlation coefficient (ρ). Correlation heatmaps were plotted using the geom_tile function in ggplot2. Analysis of similarities (ANOSIM; vegan package) was performed to compare beta diversity between pH groups for category C samples. Satellite maps of both Waimangu and Waikite geothermal fields (category B sites) were generated from Google using the ggmap package v3.0.0 (Kahle and Wickham, 2013), with latitude and longitude coordinates (WGS84) of geothermal features added *via* ggplot2. The topographic layers for the TVZ map were obtained from Toitū Te Whenua-Land Information New Zealand (LINZ; CC-BY-4.0), and the TVZ boundary was defined using data from Wilson et al. (1995).

## Results

### Relative read abundances of taxa fluctuated in some control geothermal features over time

**Figure 3:**
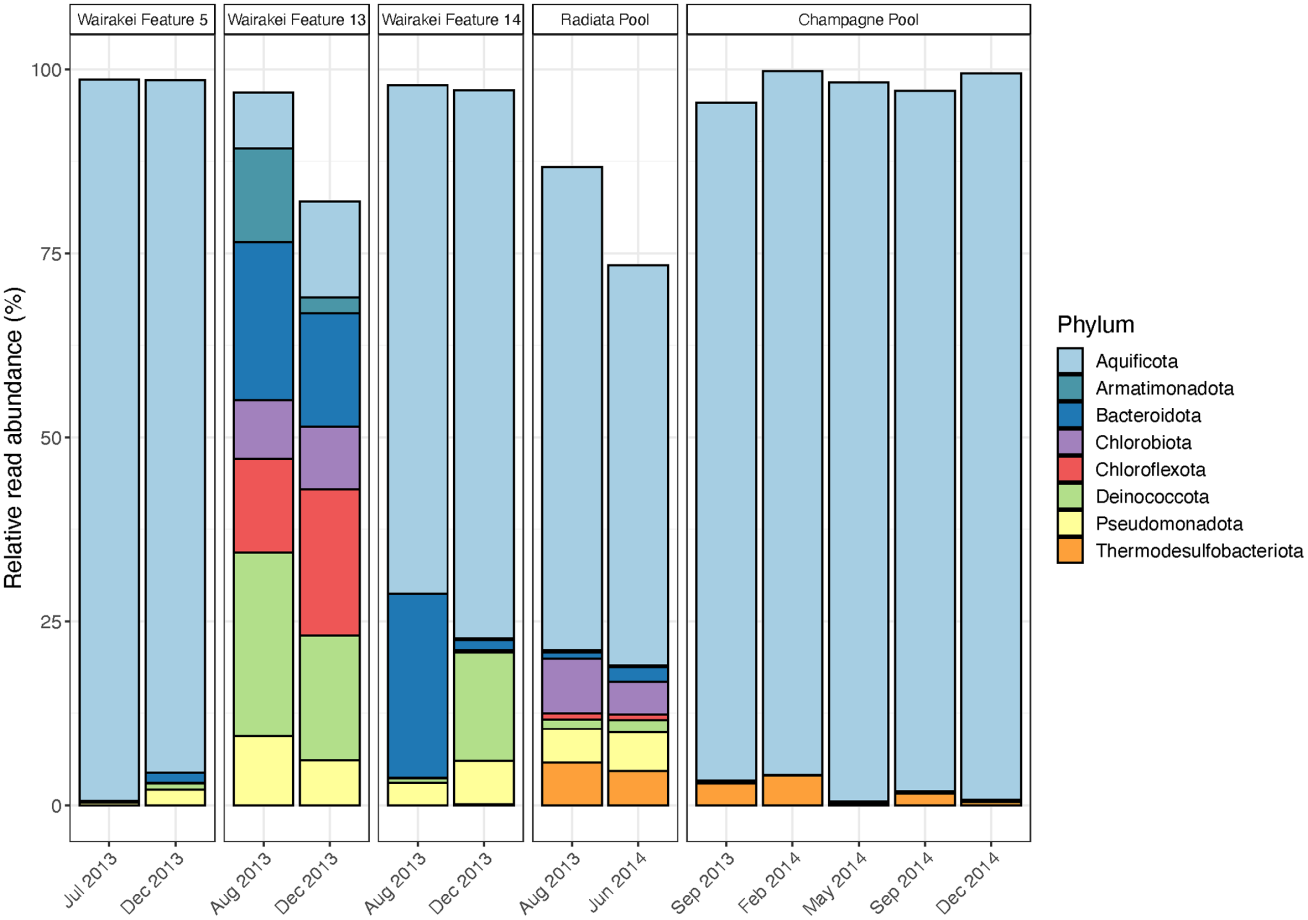
Temporal microbial community composition and relative abundance of control features in the Taupō Volcanic Zone (TVZ), Aotearoa-New Zealand. Amplicon sequencing of the 16S rRNA gene was used to measure read abundance of taxa in spring communities, with only phyla >1% average relative abundance across all samples in this category shown. Control features were sampled from the Wairakei-Tauhara (Wairakei Features 5, 13 and 14), Ngatamariki (Radiata Pool) and Waiotapu (Champagne Pool) geothermal fields.

Microbial community composition remained broadly consistent in control features throughout the duration of this study, but relative read abundances of these dominant taxa were variable in some sites (Figure 3; Supplementary Table S5). Notable abundance changes in geothermal features from the Wairakei field included increased Chloroflexota coupled with decreased Armatimonadota and Deinococcota in Feature 13, and increased Deinococcota with decreased Bacteroidota in Feature 14. These changes corresponded with temporal variation in community richness (i.e., the number of OTUs) and evenness (Shannon diversity index), while both pH and temperature remained stable for all features (Supplementary Figure S4; Supplementary Table S5). Similarly, no significant change was observed in microbial community taxa and physicochemistry of Radiata Pool, Ngatamariki, with Aquificota displaying the greatest variation to relative abundance (*SD* = 8.0%). Community structure in Champagne Pool, Waiotapu, was dominated by Aquificota in all samples, with OTU richness, evenness, pH and temperature remaining relatively constant over all seasons throughout the 15-month sampling timeframe (Supplementary Figure S4; Supplementary Table S5). Additionally, no significant correlations (*p* < 0.05, Spearman’s correlation coefficient) were observed between either alpha diversity metric with pH and temperature. Beta diversity analysis for all control features grouped samples by their respective geothermal spring (Supplementary Figure S4), indicating decreased temporal variation at a community level for these sites.

### Alpha and beta diversity remained consistent despite substantial short-term physicochemical disturbance

Evidence of a pulse disturbance in the Waimangu geothermal field was observed in microbial communities of Waimangu Stream downstream from Inferno Crater Lake overflow, with variation in relative abundances of resident taxa also occurring irrespective to this disturbance (Figure 1; Supplementary Table S6). The microbial community of Inferno Crater Lake drastically changed between timepoints, indicated by an almost complete community turnover from Pseudomonadota (72.4% of the total spring community assigned to *Acidithiobacillus* sp.) to Thermoproteota (85.6% of the total spring community assigned to Sulfolobaceae), which corresponded with an associated temperature change of 41.0 to 69.1°C for this feature during overflow (Supplementary Figure S5; Supplementary Table S6). These Thermoproteota signatures were subsequently evidenced in downstream sites of the overflow, decreasing in relative abundance from 12.5% of the total microbial community in Waimangu Feature 4 to 0.1% in Waimangu Feature 7. Other variable phyla across Waimangu Stream included increases in Pseudomonadota (not *Acidithiobacillus* sp.) in all sites except Feature 4, and decreases in Cyanobacteria in all sites. Features 6 and 7, which were near the stream outflow into Lake Rotomahana, also had increases in Bacteriodota, which corresponded with decreases in obligately thermophilic Aquificota. The effect of Inferno Crater Lake overflow was apparent in the physicochemistry of features immediately downstream of the overflow entry point (Supplementary Figure S5; Supplementary Table S6), where pH decreased from 6.7 to 3.0 in Feature 4, and 7.4 to 3.0 in Feature 5. Temperature fluctuation in downstream sites was less apparent, with the greatest temperature increase due to overflow (+6.7°C) occurring in Waimangu Feature 4. Interestingly, alpha diversity analyses did not reflect the physicochemical results, with the greatest changes observed in OTU richness of Waimangu Feature 4 and Feature 6 (Supplementary Figure S5; Supplementary Table S6). Despite a complete shift in microbial community structure in Inferno Crater Lake between the two timepoints, both alpha diversity measures for this ecosystem had negligible variation, with an associated temperature difference of 28.1°C (Supplementary Figure S5; Supplementary Table S6). The greatest dissimilarity in beta diversity between the temporal stream samples was found in Features 6 and 7 (Supplementary Figure S5). Even with the pH and temperature changes in Features 4 and 5 post-disturbance, beta diversity did not show variance between the microbial communities of these features over time.

### Diversity metrics showed initial response to long-term anthropogenic disturbance

**Figure 4:**
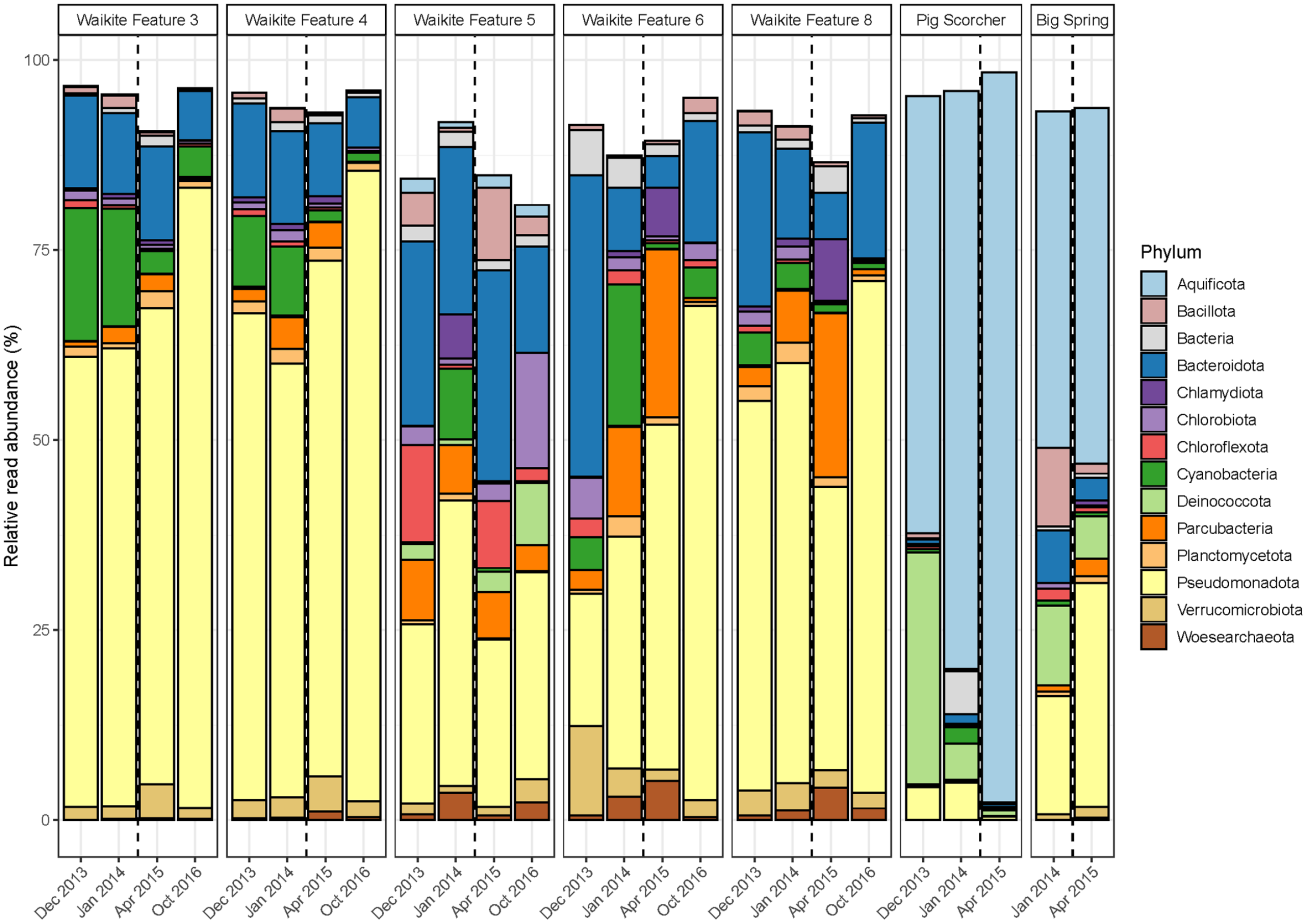
Temporal microbial community composition and relative abundance of geothermal features during the Waikite wetland restoration. Amplicon sequencing of the 16S rRNA gene was used to measure read abundance of taxa in spring communities, with only phyla >1% average relative abundance across all samples in this category shown. The vertical dashed line indicates the construction of a weir near the outlet of the wetland.

In contrast to the short-term, natural disturbance observed at Waimangu, variations in alpha and beta diversity of microbial communities did correspond with a long-term, anthropogenic disturbance at the Waikite wetland, in particular for sites located immediately before and after the installation of a weir (Features 6 and 8, respectively; Figure 2; Supplementary Table S7). In all wetland sites, OTU richness and evenness then returned to pre-disturbance levels for the final sampling timepoint (23 months post-weir installation). Beta diversity showed an initial response to the disturbance for all sites in or near Otamakokore Stream, which was then amplified for the final timepoint of Features 3, 4 and 8. Features with an additional geothermal input (Feature 5 and “Pig Scorcher”) demonstrated variability in beta diversity irrespective of the disturbance, which was also apparent in the relative abundances of community structure in these sites (Figure 4; Supplementary Table S7). For example, the “Pig Scorcher” geothermal spring had a notable decrease in Deinococcota before the disturbance was introduced to the wetland, which coincided with an increase in Aquificota. Additionally, the microbial community of the “Big Spring” exhibited fluctuations in relative phyla abundances before and after the weir installation. However, as only two timepoints were sampled for this site, the question of whether these changes were associated with the disturbance remain unresolved. While the resident taxa of microbial communities upstream to the wetland (Features 3 and 4) remained constant over time, increases in the relative abundances of Pseudomonadota, coupled with decreases in Cyanobacteria and Bacteriodota, did correspond with the weir installation (Figure 4). Similarly, the relative abundances of Pseudomonadota increased, while Bacteriodota and Verrucomicrobiota decreased in Waikite Feature 6, a site within the wetland, although compositional changes not related to the disturbance were also noted in the microbial community of this feature. The outlet to the wetland (Feature 8) showed increases in Parcubacteria and Chlamydiota at the first timepoint post-disturbance, but these reduced to similar levels as the first timepoint by the end of the experiment. Overall, Pseudomonadota increased and Bacteriodota decreased at the wetland outlet across the 3 years analysed. Regarding physicochemistry, all features in and around the wetland decreased in both pH and temperature from December 2013 to October 2016 (Figure 2; Supplementary Table S7). The majority of these changes were minor (pH: *SD* ≤ 0.2; temperature: *SD* ≤ 4.5°C), except for Waikite Features 6 and 8 which had standard deviations of pH 0.4 and 13.5°C, and pH 0.6 and 10.3°C, respectively. Despite these variations, no significant correlations (*p* < 0.05, Spearman’s coefficient) were observed between diversity and temperature for all features, with only beta diversity of Waikite Features 4 and 8 producing meaningful correlations with pH (Supplementary Figure S6).

### Temperature is more variable than pH over time in geothermal features

**Figure 5:**
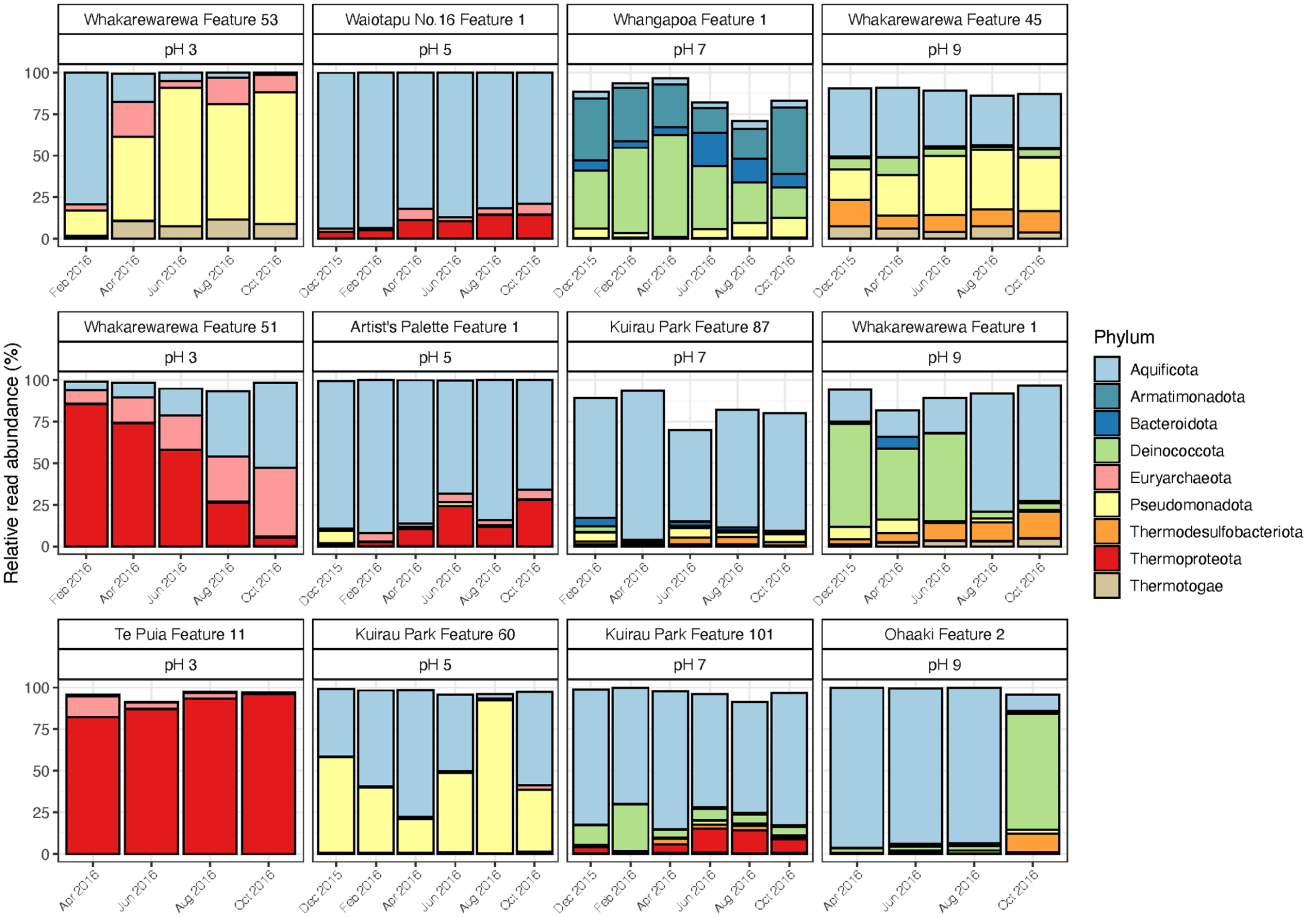
Temporal microbial community composition and relative abundance of geothermal features across the pH scale. Amplicon sequencing of the 16S rRNA gene was used to measure read abundance of taxa in spring communities, with only phyla >1% average relative abundance across all samples in this category shown. Geothermal features are grouped according to their respective pH group (pH 3, 5, 7, and 9).

Similar to control and disturbed samples, temporal variations were observed in the relative abundances of geothermal microbial communities across a range of pH values (pH 3 to 9; Figure 5; Supplementary Table S8). Six of the original 69 samples did not yield sufficient sequence reads (<5,000) after rarefaction, resulting in 63 samples for final community analysis. The greatest changes to relative abundances occurred in two features from the pH 3 group (Whakarewarewa Features 51 and 53), and two features from the pH 9 group (Whakarewarewa Feature 1 and Ohaaki Feature 2). Aquificota taxa were involved in all these changes, with relative abundance of the phylum increasing in Whakarewarewa Feature 51, but decreasing in Whakarewarewa Feature 53 over the timeline of the experiment. These variations were coupled with decreasing Thermoproteota (family Sulfolobaceae; Supplementary Figure S7) and increasing Pseudomonadota, respectively, while the balance of Aquificota in the two pH 9 features was inversely proportional to Deinococcota taxa. Aquificota was also the most abundant phylum in five of six features from the pH 5 and 7 groups (as genus *Venenivibrio*; Supplementary Figure S7), with notable variation to this phylum only occurring in Kuirau Park Feature 60. Whangapoua Feature 1 (pH 7 group) was the only feature across all 12 sites in this category to present change independent of Aquificota; here, relative abundances of Armatimonadota, Bacteroidota and Deinococcota fluctuated over the 10-months analysed.

**Figure 6:**
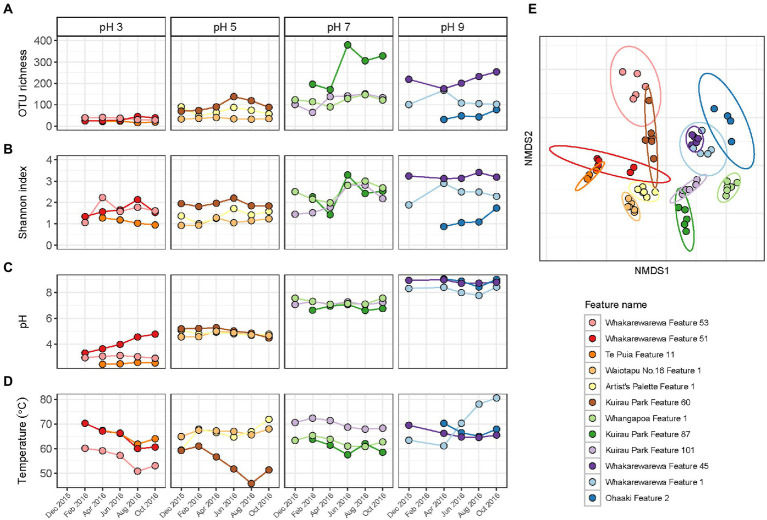
Temporal diversity and physicochemistry of geothermal features from across the pH range. **(A)** The microbial communities of 12 geothermal springs were measured six times over 1 year by amplicon sequencing of the 16S rRNA gene, with variation in alpha diversity between temporal samples indicated by OTU richness (i.e., the number of OTUs per community). **(B)** Alpha diversity was also measured by the Shannon diversity index to indicate evenness of the communities over time. **(C)** Spring pH of the features at the time of sampling. **(D)** Spring temperature of the geothermal features. **(E)** Beta diversity of the 12 features is shown by a non-metric multidimensional scaling (NMDS; *n* = 62, stress = 0.15) of Bray-Curtis dissimilarities between all temporal samples, with data ellipses generated from multivariate t-distribution with a 95% confidence level. All samples are coloured according to feature name, with alpha diversity and physicochemistry further grouped by pH group.

Alpha and beta diversity metrics for all 12 features in this category produced contradictory results (Figure 6; Supplementary Table S8), with the greatest variation in alpha diversity presented in the pH 7 group features. Conversely, beta diversity suggested geothermal features from pH 3, 5 and 9 groups were more likely to have varied community structure over time, and were more representative of the variation in relative abundances than alpha diversity. pH remained relatively stable over time for the majority of features, with only Whakarewarewa Feature 51 (pH 3 group) having a standard deviation >0.3 pH units (Figure 6; Supplementary Table S8). Analysis of similarities confirmed that variation in beta diversity was greater between pH groups than within pH groups (*p* = 0.001, ANOSIM; Supplementary Figure S8), indicating the significant influence of pH on driving diversity in these ecosystems on a spatial scale. However, temperature exhibited a greater temporal variability than pH, with only four features having a standard deviation of ≤2°C (Figure 6; Supplementary Table S8). Whakarewarewa Feature 1 (pH 9 group) had the greatest fluctuation in temperature overall, changing from 63.4 to 80.6°C, over the sampling period. The effect of temperature on these ecosystems was highlighted by six features producing significant positive correlations between temperature and beta diversity (*p* < 0.05, Spearman’s coefficient; Supplementary Figure S9). The physicochemical relationship with alpha diversity was not as conclusive, with only four features demonstrating a significant correlation between temperature and either OTU richness or evenness (*p* ≤ 0.03, Spearman’s coefficient; Supplementary Figure S9). Corresponding with the pH stability we observed in these geothermal features, Whakarewarewa Feature 51 (group pH 3) was the only feature to produce a positive correlation between pH with both alpha and beta diversity metrics (OTU richness, ρ = 0.70, *p* = 0.03; Bray-Curtis dissimilarities, ρ = 0.84, *p* = 0.01; Spearman’s correlation coefficient).

## Discussion

Microbial community analysis of 31 geothermal features from the Taupō Volcanic Zone, Aotearoa-New Zealand, suggests abundances of taxa can vary stochastically over time due to a complex confluence of both apparent and cryptic parameters, with a sustained physicochemical change necessary to provoke extensive community turnover. Conversely, the composition of microbial taxa did not change in the five features sampled temporally as control sites (Figure 3), with physicochemistry also remaining stable (Supplementary Figure S4). Of these control sites, Wairakei Feature 5 and Champagne Pool displayed the least variation in relative abundances. These sites are both replenished *via* deeply-sourced hydrothermal fluids that exhibit limited interaction with groundwater (Bignall et al., 2010; Gallagher et al., 2020). The rate of physicochemical change for these fluids would be much slower than other inputs to shallower (i.e., acidic) geothermal springs, such as meteoric and/or groundwater, which are more readily affected by the level of the water table and atmospheric events (Campbell et al., 2017; Colman et al., 2021). Thus, the microbial communities of geothermal features with deeply sourced hydrothermal fluids are sustained by a more constant environmental niche, which consequently promotes a stable ecosystem structure. The physical structure of a geothermal feature is an important attribute to note, as it has been proposed that smaller springs are more susceptible to environmental change (Campbell et al., 2017). While diurnal variation to the physicochemistry of Champagne Pool was suggested to be a consequence of probable microbial activity (Pope et al., 2004; Ullrich et al., 2013), our findings indicate that microbial community abundances of this large geothermal spring are unaffected by seasonality and weather events. These results are consistent with previous research on the lack of microbial community variation across seasons in geothermal waters (Siering et al., 2013; Mitrović et al., 2022) and reinforce the concept that source fluids for geothermal springs (i.e., from either near-surface or deep hydrothermal aquifers) regulate the influence of seasonality on community assemblages (Colman et al., 2021). The inferred dogma for non-extreme environments is also supported; espousing a stable environment encourages low turnover of dominant taxa in resident microbial communities (Nguyen et al., 2021), with a disturbance that alters major physicochemical conditions required to shift community structures at the phylum level.

While analysis of microbial community response to both natural and anthropogenic disturbances corroborated fluctuation in abundances of taxa irrespective of measured environmental change, variation to both taxa composition and relative abundance were noted in geothermal features in the immediate vicinity of perturbations. The influence of Inferno Crater Lake, as evidenced by the dominant population of Thermoproteota (family Sulfolobaceae) and physicochemical conditions during overflow (Figure 1), was clearly apparent in Waimangu Stream sites downstream of the pulse disturbance. The pH of the stream was more readily affected than temperature during this coalescence (pH 6.7 to 3.0, and 49.9 to 56.6°C; Waimangu Feature 4; Supplementary Figure S5), indicating minimal buffering capacity of the stream water; with the volumetric rate of Inferno Crater Lake overflow (~79 l s^−1^) only marginally increasing the temperature of the stream. However, these physicochemical changes to Waimangu Stream are not conserved as the Inferno Crater Lake overflow is short-lived (2–3 days; Ward et al., 2017). With microbial communities in the stream experiencing this disturbance every 30–40 days, we now know resident populations quickly revert back to pre-disturbed states; the previous overflow event to this study ended 8 days prior to sampling (Supplementary Figure S3). Waimangu Stream (~104 l s^−1^) provides a constant reservoir of microbial populations from Frying Pan Lake that can quickly re-colonise downstream sites once the disturbance has ended, thereby providing a short residence time for the disturbed communities (Jones et al., 2020). Frying Pan Lake also undergoes reservoir cycling, albeit less extreme than Inferno Crater Lake (Scott, 1994), which could explain the alternating abundances of both Cyanobacteria and Pseudomonadota observed in all stream sites. Similar to reports on freshwater microbial assemblages (Shade et al., 2012; Simon et al., 2016), these findings suggest geothermal stream communities are resilient and can return to a pre-disturbance state if physicochemical conditions stabilise, and sufficient re-colonisation is attainable. However, microbial variation in Waimangu Stream and Inferno Crater Lake was not clear in alpha and beta diversity measures, with the greatest temporal dissimilarity in beta diversity occurring in the two sites furthest away from the disturbance (Supplementary Figure S5). These features could be influenced by other geothermal springs not associated with upstream samples, and/or proximity to the stream outflow into Lake Rotomahana likely facilitates lacustrine water inputs to these ecosystems, supported by an observed increase in Bacteroidota which are commonly found in freshwater lake epilimnia (Newton et al., 2011). Additionally, 16S rRNA gene sequencing may not capture the total diversity of any microbial community, therefore all changes to resident populations might not have been captured. Nevertheless, even though phylum-level changes occurred in stream communities immediately downstream of the coalescence disturbance point, these short-term variances were not reflected in diversity analyses.

Conversely, the long-term disturbance at the Waikite geothermal field was most evident in the beta diversity of temporal microbial communities at the outlet to the wetland, which corresponded with the greatest variation in physicochemistry of all sites studied in this area (Figure 2). Even though there was a temperature decrease of 20°C at the outlet, diversity only correlated with a decrease in pH from 8.6 to 7.6, indicating that a larger magnitude of change in temperature may be required than pH to influence microbial populations in geothermal ecosystems. Interestingly, alpha diversity of all stream sites around the wetland showed increases in diversity in the first timepoint post-disturbance, but these had returned to pre-disturbance levels by the final timepoint (Figure 2). These observations advocate the importance of using multiple diversity metrics to investigate microbial communities over time so that a cohesive picture of ecosystem behaviour is obtained (Wagner et al., 2018). Both relative abundances and beta diversity of microbial communities within (Features 5 and 6) and outside the wetland (‘Pig Scorcher’ and ‘Big Spring’) fluctuated irrespectively of the perturbation (Figure 4), with no correlation to pH or temperature (Supplementary Figure S6). Features 5 and 6 have additional geothermal inputs to Otamakokore Stream, and are relatively low volume features, suggesting they are more susceptible to fluctuation in environmental conditions (Campbell et al., 2017). A notable temperature difference (93.8 to 87.2°C) was observed for the ‘Pig Scorcher’ spring after the weir installation, however, this physicochemical change was not attributed to wetland dynamics (Reeves et al., 2018). It should be noted that both sampling timepoints after the disturbance occurred outside of the austral summer, which could partially explain the decrease observed in Cyanobacteria at all wetland sites. Even though limited Cyanobacterial change was reported with or without ultraviolet radiation in geothermal microbial mats (Norris et al., 2002), seasonal variation was detected in other geothermally associated Cyanobacteria populations between dry and rainy seasons (Lacap et al., 2007). Greater temporal variation can occur in the microbial communities of geothermal sediments than associated water columns (Wang et al., 2014), and with shorter residence times for taxa in stream sites than other geothermal features, physical processes enabling community assembly through time would conceivably alternate across sample types. Overall, these findings suggest the abundances of planktonic microbial communities acclimatise to sustained physicochemical change in the local environment, but there is a general instability or hysteresis in geothermal habitats, even when physicochemistry appears relatively stable.

Similar to the previous categories of geothermal features, relative abundances of microbial communities from all pH groups varied over the 1-year sampling timeframe (Figure 5), with temperature fluctuating more than pH in these habitats (Figure 6). The pH 7 group had the most change in alpha diversity, particularly for Shannon diversity index (Figure 6), which could be a result of the increased number of taxa that prefer circumneutral conditions (Sharp et al., 2014). In contrast, the greatest changes in beta diversity were detected in pH 3, 5 and 9 groups (Figure 6), which corresponded with the variation observed in relative abundances of taxa in these features. Like microbial community analysis of Inferno Crater Lake, these results suggest alpha diversity fails to adequately measure temporal variation outside of circumneutral pH. This could be due in part to reduced diversity potential to fill any voids created by changing communities at either ends of the pH spectrum for geothermal springs, with the magnitude change that occurs with varying pH outside of circumneutral being more impactful. Previous longitudinal research has suggested alpha diversity can fail to present change despite highly divergent microbial communities (Wagner et al., 2018), again reinforcing the use of multiple diversity metrics when investigating temporal scales. Whakarewarewa Feature 51 (pH 3 group) was the only feature that significantly correlated with pH for both alpha and beta diversity metrics (Supplementary Figure S9), with a corresponding pH increase of 3.3 to 4.8 observed. This feature also had a positive correlation between diversity and temperature, along with six other features from across the pH range (Supplementary Figure S9), suggesting temperature is more likely to fluctuate over time than pH in geothermal features. At least eight of the features in this category are shallow (<2 m in depth), and so are more susceptible to meteoric and groundwater fluctuations that occur seasonally in Aotearoa-New Zealand (Zheng and Frederiksen, 2006).

Comparable to the microbial community of Inferno Crater Lake, Thermoproteota populations (family Sulfolobaceae) decreased in Whakarewarewa Feature 51 as the temperature decreased and pH increased over the study period (Figure 6; Supplementary Figure S7). This population decrease, characteristic to Sulfolobaceae when pH increases (Albers and Siebers, 2014), was counteracted with increased Aquificota, and indeed, Aquificota featured prominently in geothermal community fluctuations from across the pH scale. This could be a result of the widespread abundance of this phylum found throughout the TVZ (Power et al., 2018), but temporal changes to Aquificota populations have also been observed in other geothermal systems worldwide (Briggs et al., 2014; Ward et al., 2017; Colman et al., 2021), suggesting hypersensitivity in this phylum to physicochemical change. Interestingly, Ohaaki Feature 2 (pH 9 group) had an almost complete community shift from Aquificota to Deinococcota (Figure 5), which correlated with both groundwater and hydrogen sulphide levels. Hydrogen sulphide gas frequently percolates through geothermally-heated soil in the TVZ (Chambefort, 2021), and consequently is common to geothermal springs that are influenced by groundwater. Therefore, this observed shift indicates water table levels directly affect the microbiology of this spring. We also know that historic modification, stemming from local geothermal power generation, influenced both temperature and water levels of the spring (Glover et al., 1996), so anthropogenic impacts could explain the community variation observed in this study. Regardless, geothermal features from a range of source fluids across the pH scale exhibited both stochastic and deterministic fluctuation in the relative abundances of microbial communities, proposing that all geothermal features do not adhere to the same processes of community assembly through time.

## Summary

Our study of 31 individual features indicates that taxa abundances fluctuate temporally in geothermal microbial communities; with stochastic or unexplained variation still occurring across a wide pH range. Temperature is more variable than pH over time in these habitats, however, community assemblages appear more sensitive to changes in pH than temperature. Geothermal features with a deeply-sourced fluid input are less susceptible to physicochemical change and consequently have a more stable microbial community; suggesting source fluid (either hydrothermal, meteoric or a mixture of both) as a main driver of temporal variation. Our unique dataset also infers geothermal ecosystems can recover from short-term physicochemical disturbances, but how we define change is pertinent as multiple diversity metrics produced disparate results over the breadth of feature categories analysed, implying these measures are not always representative of change.

There are limitations to this study that must be considered, such as focusing on large shifts in the relative abundance of phyla. Even though a recent report indicated dominant taxa are more susceptible to a changing environment than rare taxa (Kurm et al., 2019), it is likely that environmental changes actively select for finer-scale phenotypic or metabolic traits not detectable *via* the taxonomic analyses undertaken in this study (Poretsky et al., 2014). Focusing on genome-wide analysis could also infer other factors beyond physicochemistry than induce temporal change in thermophilic taxa (Campbell et al., 2017). Additionally, the reliance on diversity metrics (Shade, 2017) and the use of relative over absolute abundances (Props et al., 2017) as a proxy for designating change should be taken into account. While the effect of traditional biases, such as primer design, DNA extraction, and sequencing (Tremblay et al., 2015; Wagner Mackenzie et al., 2015; Walters et al., 2015; Wear et al., 2018), was minimised by the use of identical methodologies for processing of all samples, our study highlights the importance of using both replicates and increased sampling frequency in order to obtain a true consensus on core geothermal microbial communities.

## Data availability statement

The datasets presented in this study can be found in online repositories. The names of the repository/repositories and accession number(s) can be found at:

https://www.ebi.ac.uk/ena, PRJEB55115; https://www.ebi.ac.uk/ena, PRJEB24353. All code used for data analysis and figures is available through GitLab (https://gitlab.com/morganlab/collaboration-1000Springs/1000Springs).

## Author contributions

JP, MS, SC, and IM conceived and supported the study. JP, MS, SC, and CC contributed to experimental design. JP, CL, CC, and MS performed fieldwork. JP and CL processed samples. JP conducted bioinformatic and statistical analyses. JP, MS, and CC wrote the manuscript, with assistance from IM and SC. All authors contributed to the article and approved the submitted version.

## Funding

This research was supported by a Smart Ideas grant (C05X1203 – Microbial Bioinventory of Geothermal Ecosystems), colloquially known as the 1000 Springs Project (https://1000springs.org.nz), which was awarded by the Ministry of Business, Innovation and Employment (MBIE) of the Aotearoa-New Zealand Government to MS and SC. JP was also supported by a Te Pū Ao-GNS Science Postgraduate Scholarship under the Geothermal Resources of New Zealand (GRN) programme, and the Te Whare Wānanga o Waikato-University of Waikato Hilary Jolly Memorial Scholarship for freshwater ecology research in Aotearoa-New Zealand.

## Conflict of interest

The authors declare that the research was conducted in the absence of any commercial or financial relationships that could be construed as a potential conflict of interest.

## Publisher’s note

All claims expressed in this article are solely those of the authors and do not necessarily represent those of their affiliated organizations, or those of the publisher, the editors and the reviewers. Any product that may be evaluated in this article, or claim that may be made by its manufacturer, is not guaranteed or endorsed by the publisher.
