# Supplementary material for: Temporal dynamics of geothermal microbial communities in Aotearoa-New Zealand
Source: Front Microbiol. 2023 Mar 15;14:1094311. doi: 10.3389/fmicb.2023.1094311 (PMC10068964; doi:10.3389/fmicb.2023.1094311)
Supplement: Supplementary file 1 [file Data_Sheet_1.pdf]

SUPPLEMENTARY INFORMATION

Power, J. F. et al. Temporal dynamics of geothermal microbial ecosystems in Aotearoa-New Zealand (2023).

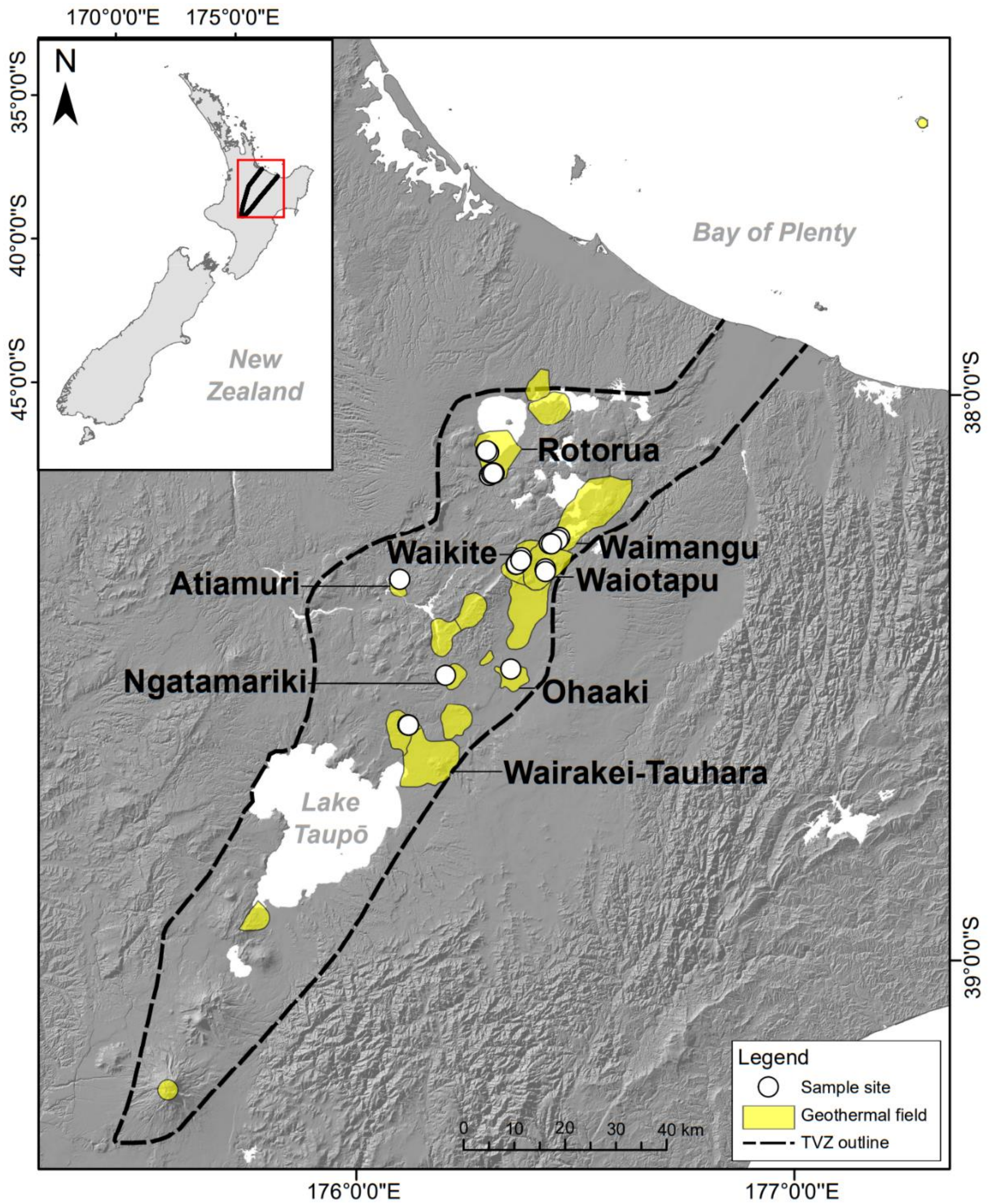

Figure S1. Map of the Taupō Volcanic Zone (TVZ), Aotearoa-New Zealand. Geothermal fields are presented in yellow, with fields used in this study labelled. Geothermal features sampled temporally are highlighted by white circles (*n*=31). The panel insert displays the location of the TVZ in the central North Island of Aotearoa-New Zealand. The topographic layers for this map were obtained from Land Information New Zealand (LINZ; CC-BY-4.0) and the TVZ boundary defined using data from Wilson *et al*., 1995.

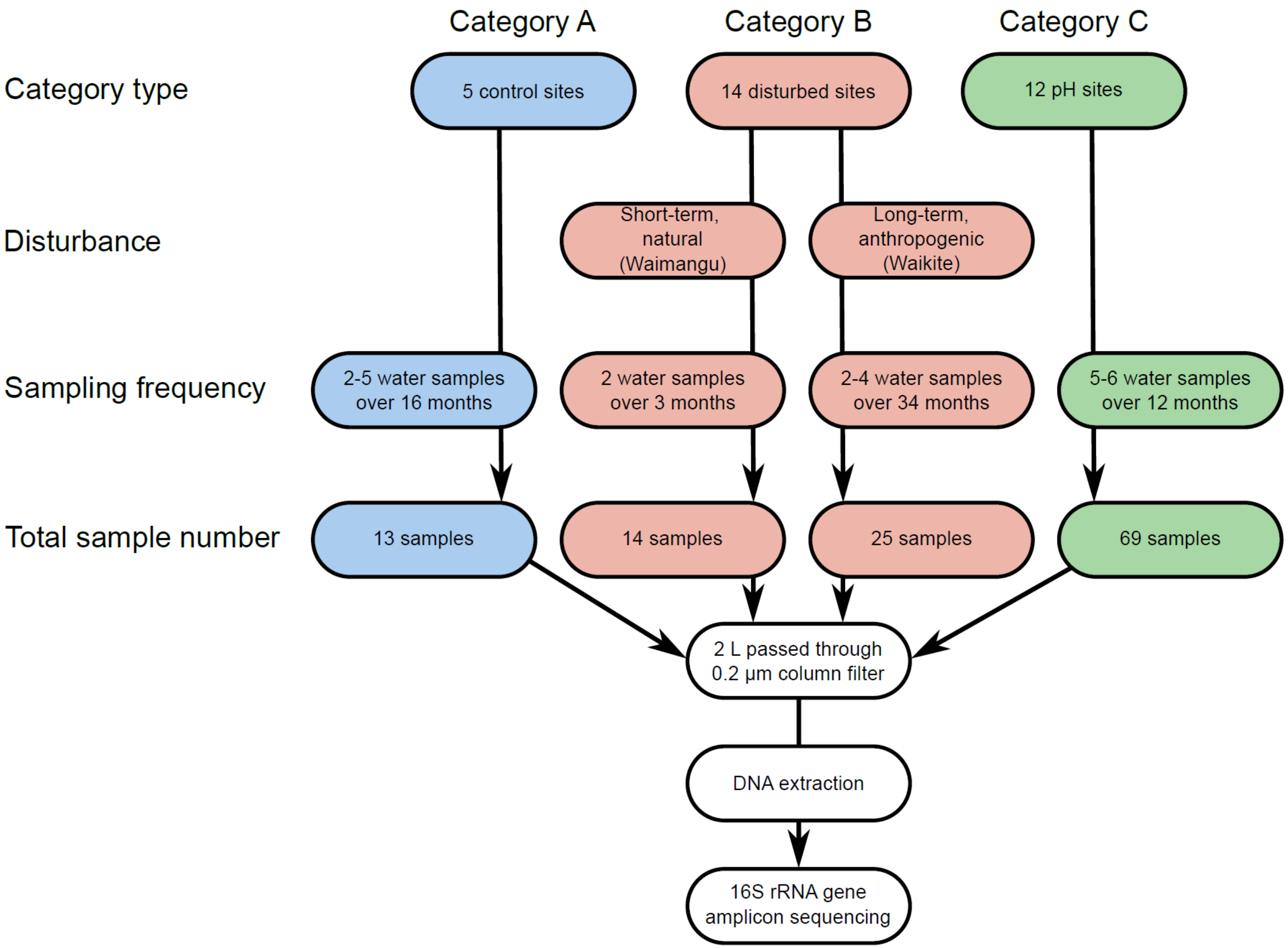

Figure S2. Schematic overview of temporal site categories, sampling frequency, and sample processing. Three categories of geothermal features are displayed in colour; control sites (category A; blue), disturbed sites (category B; red), and pH sites (category C; green). The disturbed sites were additionally split into two groups to allow study of both a short-term, natural disturbance and a long-term, anthropogenic disturbance to the microbial communities at Waimangu and Waikite geothermal fields, respectively.

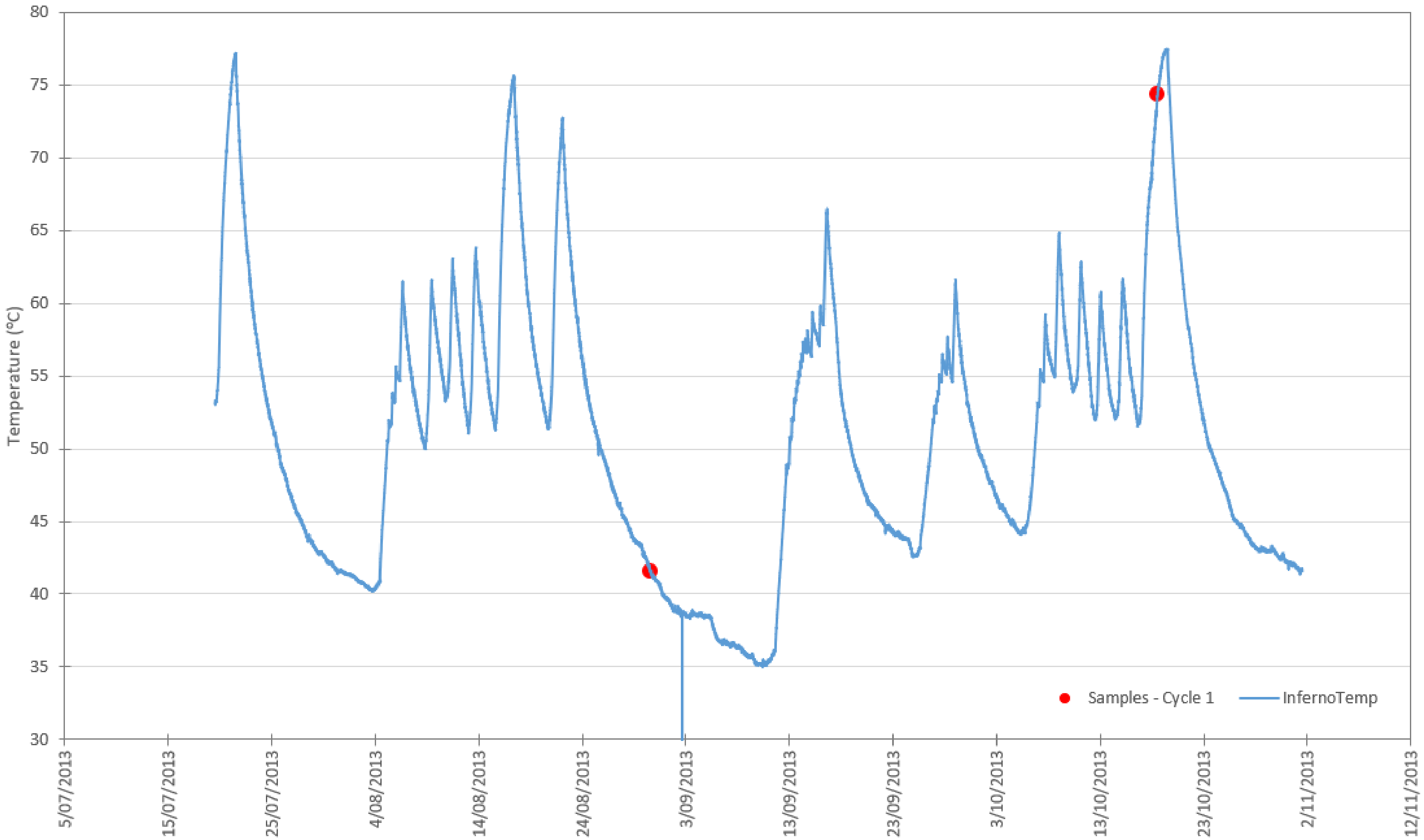

Figure S3. Temperature profile of Inferno Crater Lake. The ~30-40 day cycle of Inferno Crater Lake in the Waimangu geothermal field is indicated by the rise and fall of temperature on the y-axis, with overflow events into Waimangu Stream occuring when lake temperatures reached >70 °C. Timepoints of samples taken for this study are represented in red.

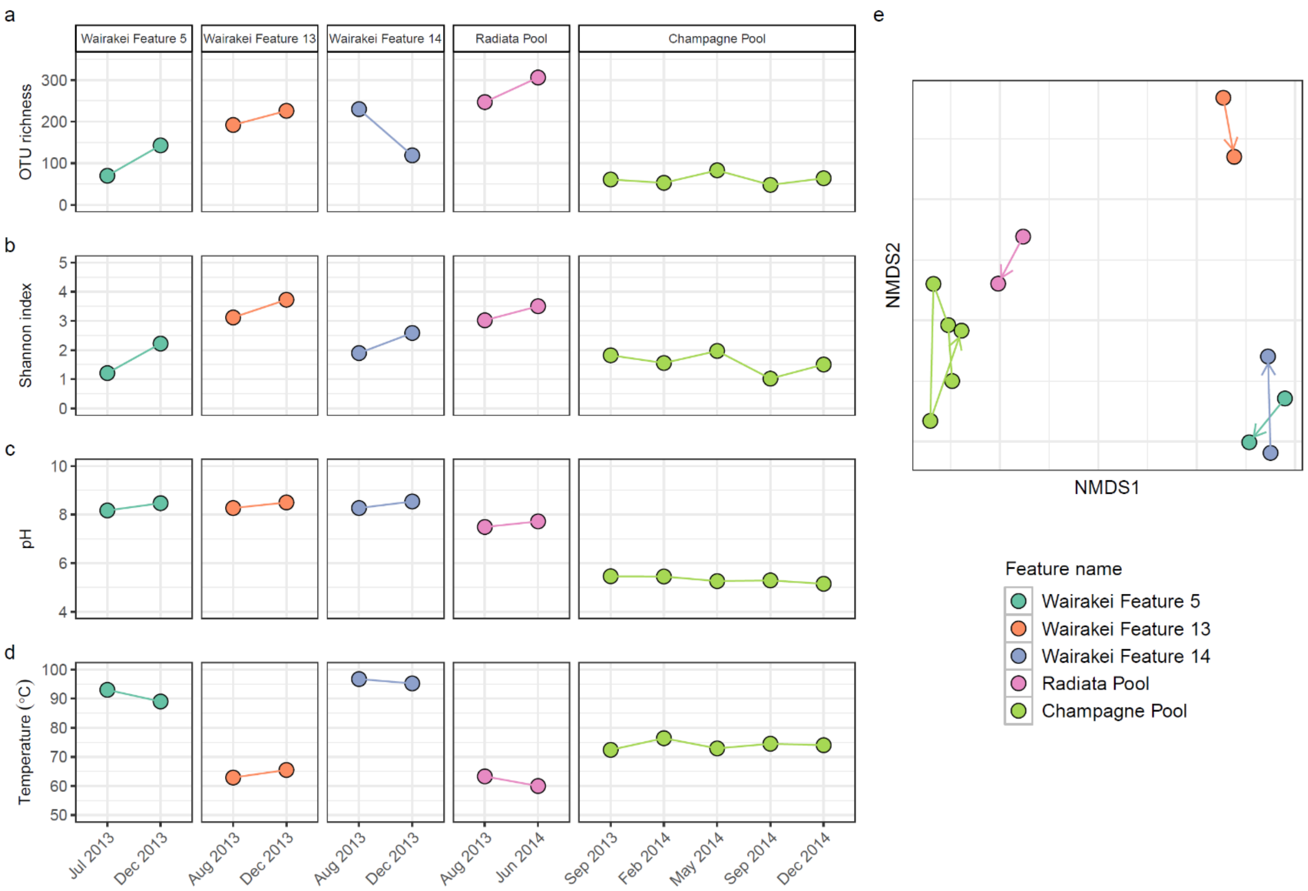

Figure S4. Temporal diversity and physicochemistry of control features in the Taupō Volcanic Zone (TVZ), Aotearoa-New Zealand. (a) The microbial communities of five control features were measured by amplicon sequencing of the 16S rRNA gene, with variation in alpha diversity between temporal samples indicated by OTU richness (i.e., the number of OTUs per community). **(b)** Alpha diversity was also measured by the Shannon diversity index to indicate evenness of the communities over time. **(c)** Spring pH of the features at the time of sampling. **(d)** Spring temperature of the control features. **(e)** Beta diversity of all five control features, distinguished by colour, is shown by a non-metric multidimensional scaling (NMDS; *n*=13, stress=0.1) of Bray-Curtis dissimilarities between samples. The timeline of sampling is indicated by the direction of the lines and arrows.

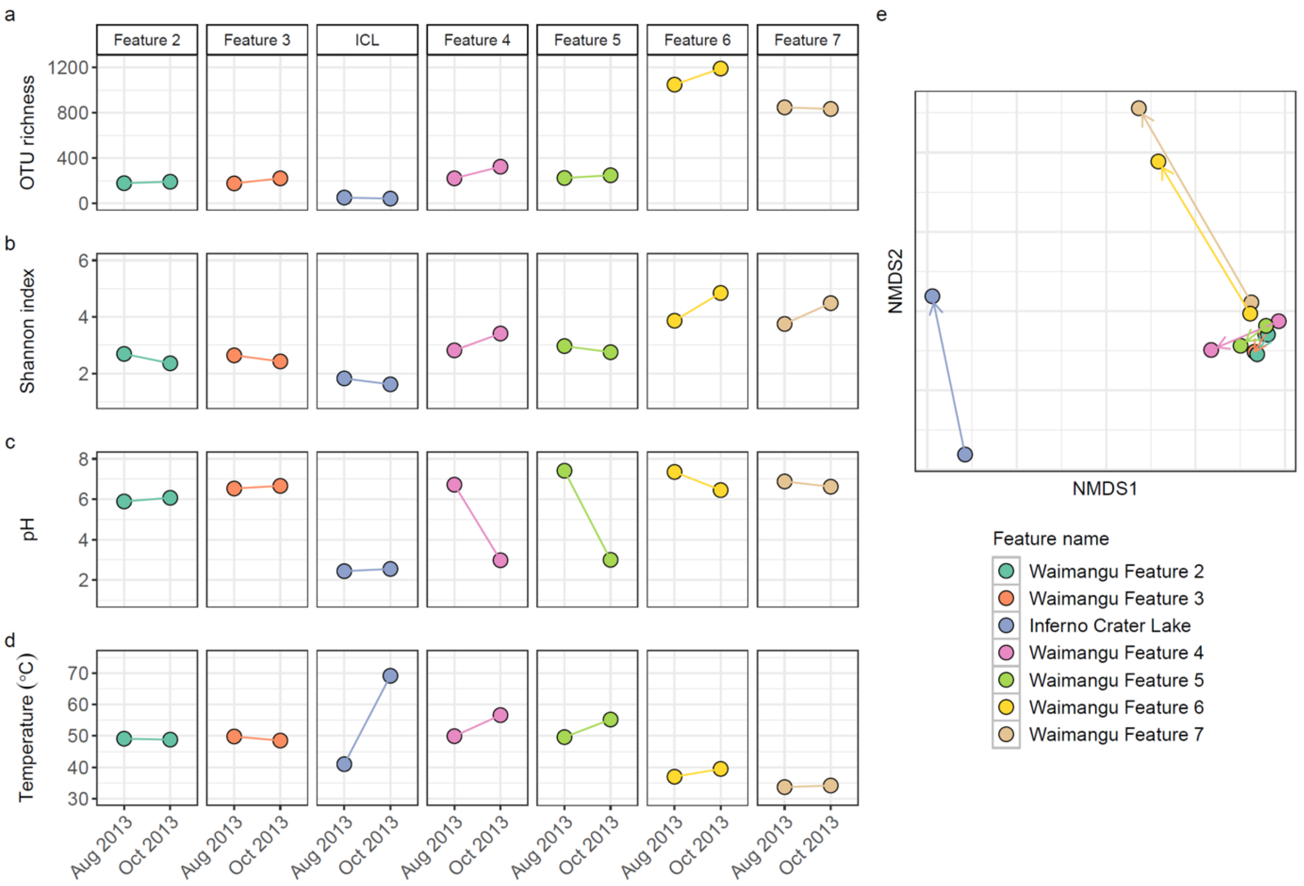

Figure S5. Temporal diversity and physicochemistry of disturbed features at Waimangu geothermal field. (a) The microbial communities of Waimangu Stream sites and Inferno Crater Lake (ICL) were measured by amplicon sequencing of the 16S rRNA gene, with variation in alpha diversity between samples indicated by OTU richness (i.e., the number of OTUs per community). **(b)** Alpha diversity was also measured by the Shannon diversity index to indicate evenness of the communities over time. **(c)** The pH of features at the time of sampling. **(d)** Temperature of both stream sites and Inferno Crater Lake. **(e)** Bray-Curtis dissimilarity was calculated for beta diversity, with nonmetric-multidimensional scaling (NMDS; n=14, stress=0.09) used to ordinate the samples in 2-dimensional space. Samples were taken before (August 2013) and during (October 2013) the overflow of Inferno Crater Lake into Waimangu Stream, with time indicated by arrows.

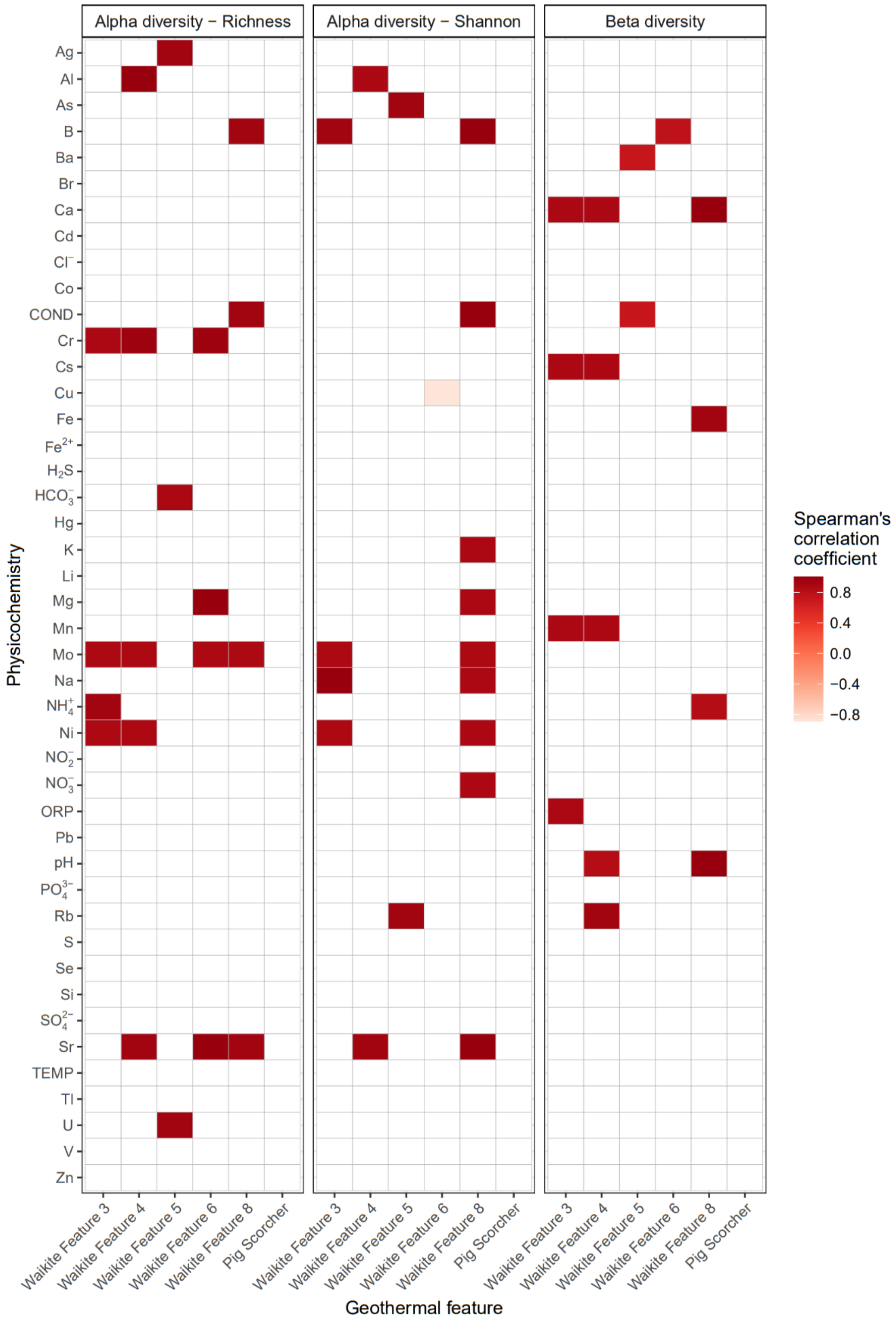

Figure S6. Relationship between diversity and physicochemistry of Waikite geothermal features. Diversity measures were calculated from amplicon sequencing of the 16S rRNA gene found in the microbial communities of these features over time. Alpha diversity was measured by both OTU richness (i.e., the number of OTUs in a community), and Shannon index (i.e., evenness of a community), with beta diversity determined by Bray-Curtis dissimilarities between temporal sites. Significant correlations (p<0.05) via Spearman's coefficient with all 44 physicochemical variables are displayed in colour.

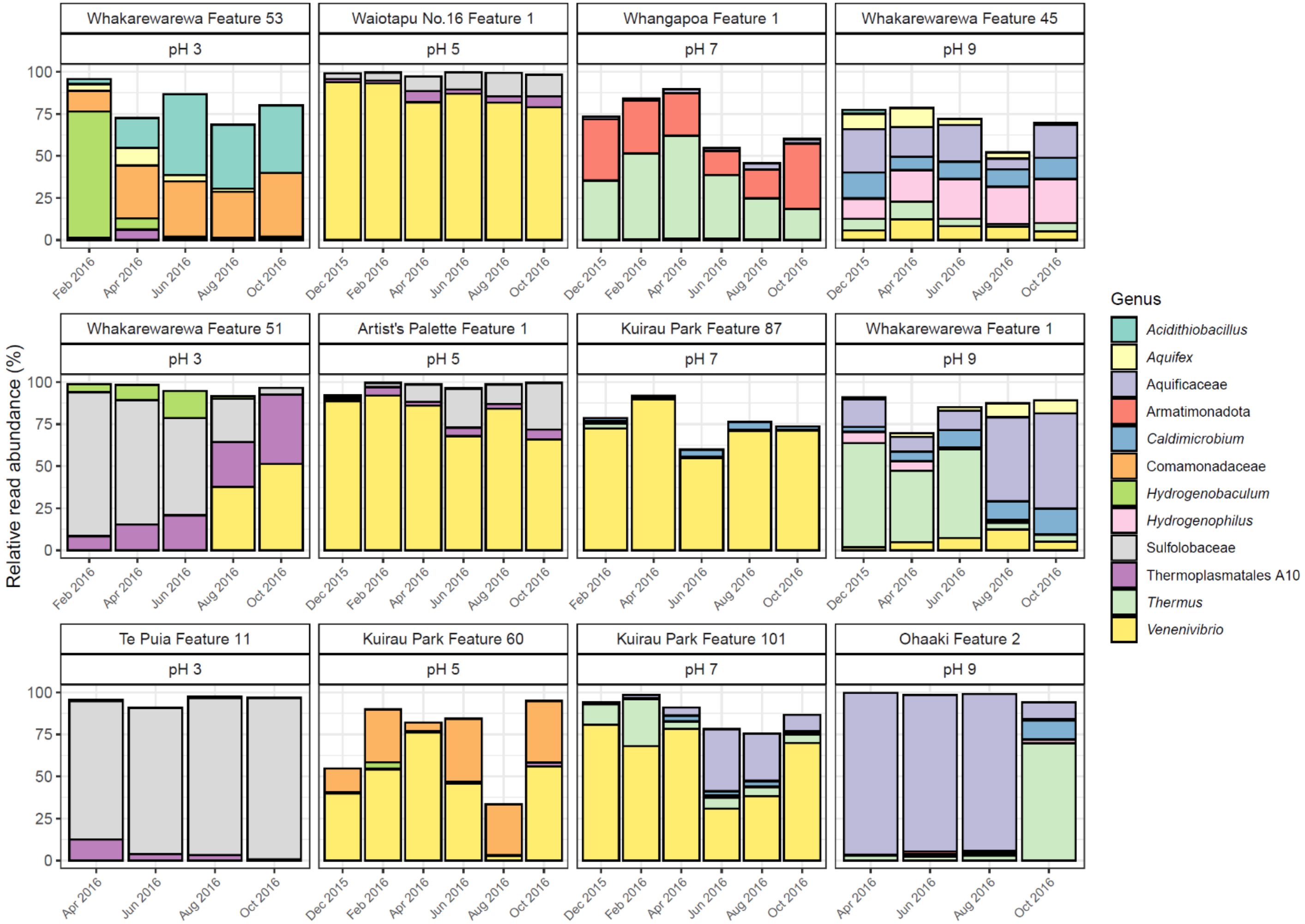

Figure S7. Temporal community composition and relative abundance of genera in geothermal features across the pH scale. Amplicon sequencing of the 16S rRNA gene was used to measure read abundance of taxa in spring communities, with only genera >1 % average relative abundance across all samples shown. Geothermal features are grouped according to their respective pH group (pH 3, 5, 7 and 9). Where taxonomy failed to classify to genus, the next corresponding taxonomic rank is displayed.

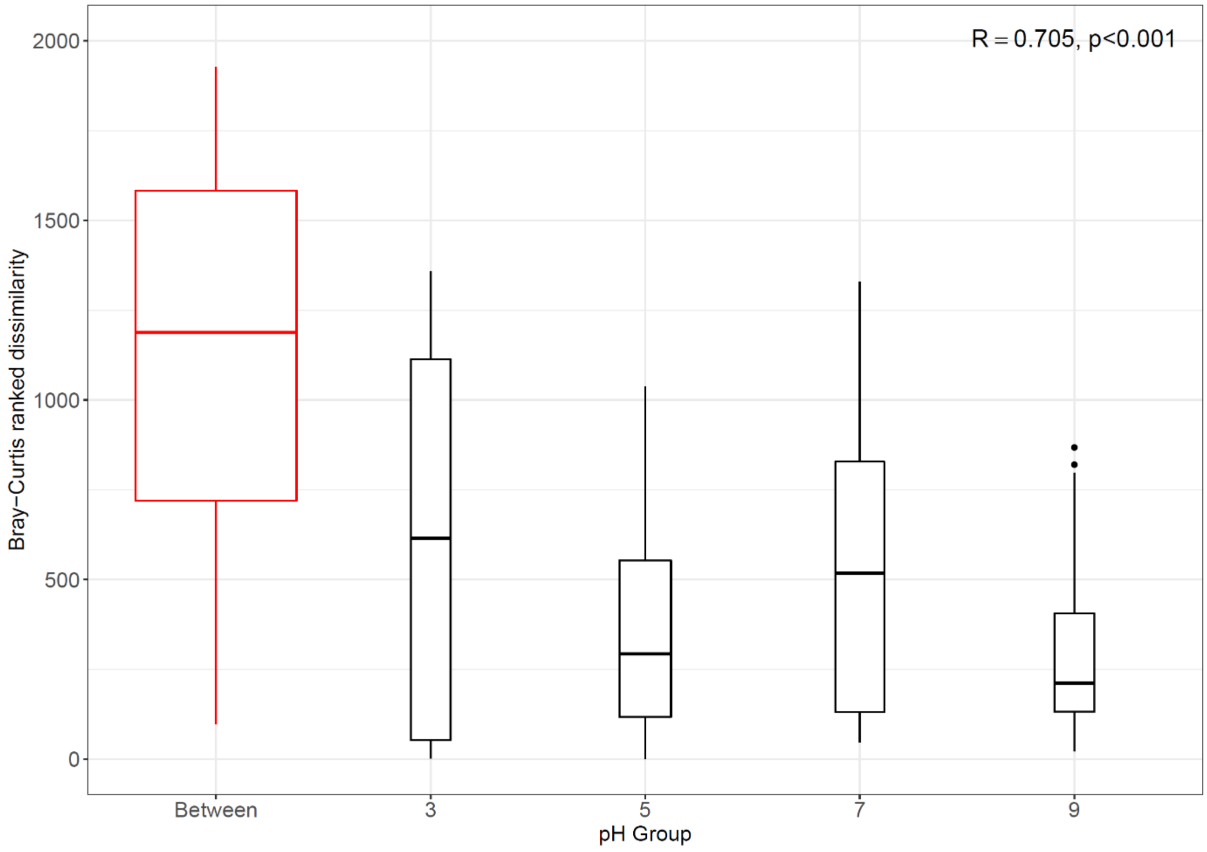

Figure S8. Beta diversity comparison of temporal samples grouped to pH. Bray-Curtis dissimilarity was used to calculate beta diversity from 16S rRNA gene sequencing of samples (*n*=63) from across the pH range found in Aotearoa-New Zealand geothermal features. Samples were binned according to pH group (3, 5, 7 and 9), with analysis of similarities (ANOSIM) demonstrating that microbial diversity between pH groups (red) was greater that the diversity within individual groups (R=0.705, $p<0.001$).

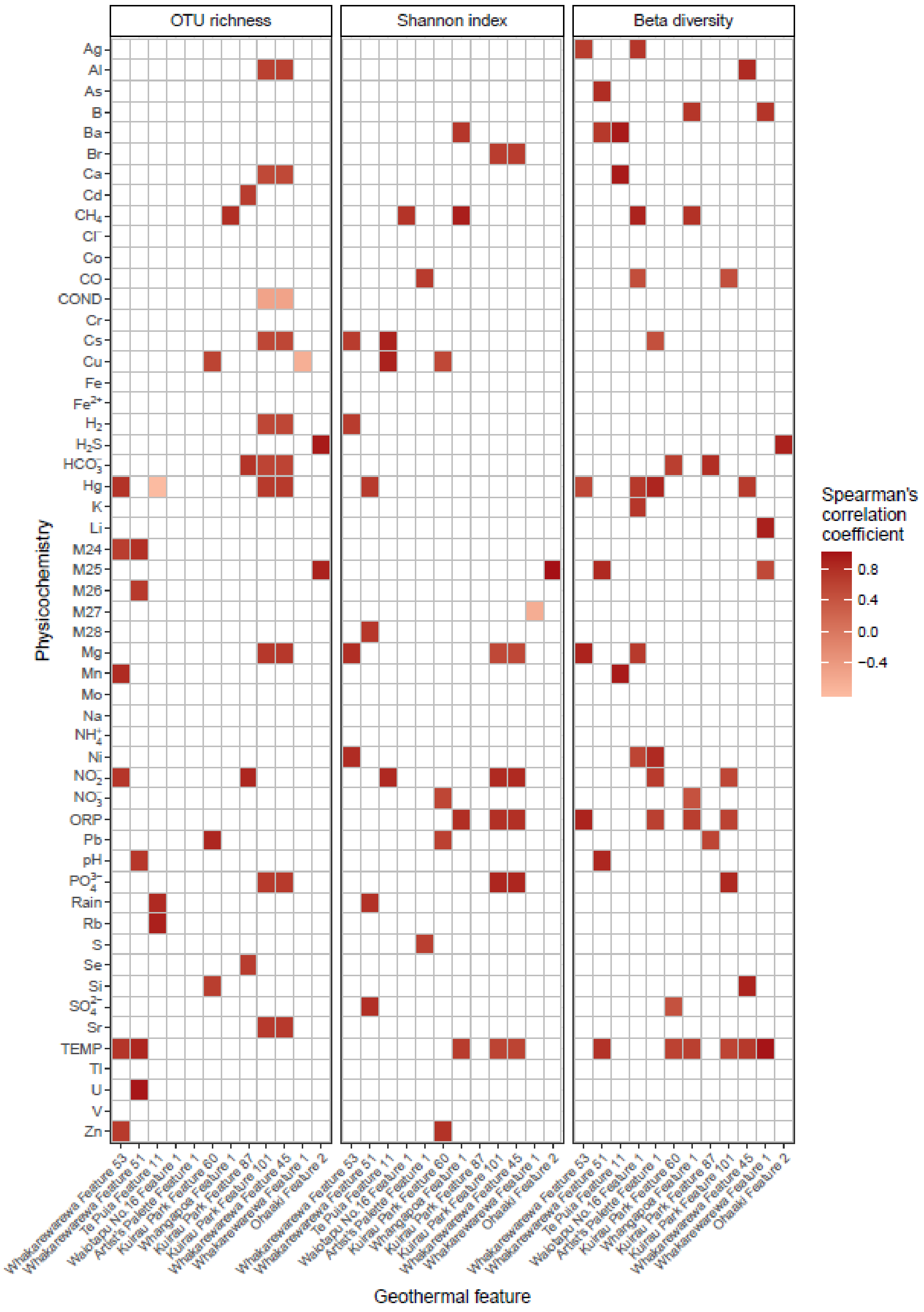

Figure S9. Relationship between diversity and physicochemistry of geothermal features across the pH range. Diversity measures were calculated from amplicon sequencing of the 16S rRNA gene found in the microbial communities of these features over time. Alpha diversity was measured by both OTU richness (i.e., the number of OTUs in a community), and Shannon index (i.e., evenness of a community), with beta diversity determined by Bray-Curtis dissimilarities between temporal sites. Significant correlations (p<0.05) with all 49 physicochemical variables are displayed in colour. Four features had positive correlations between alpha diversity and temperature (ρ≥0.57, p≤0.03, Spearman's coefficient), while the six features that had significant positive correlations between beta diversity and temperature (ρ=0.56-0.98, p<0.05) were Whakarewarewa Feature 51, Kuirau Park Feature 60, Whangapoa Feature 1, Kuirau Park Feature 101, Whakarewarewa Feature 45, and Whakarewarewa Feature 1.

Table S1. Control geothermal features and associated metadata. Alpha diversity is represented by OTU richness (i.e., number of OTUs) and evenness (i.e., Shannon index) measured in these ecosystems via 16S rRNA gene amplicon sequencing. Accessions for sample sequences are also included.

Sample ID	Study accession	Run accession	Feature name	Geothermal field	Date	pH	Temp (°C)	OTU Richness	Shannon
P1.0004	PRJEB24353	ERR2240132	Wairakei Terraces Feature 5	Wairakei-Tauhara	Jul 2013	8.2	93.0	70	1.2
P1.0140	PRJEB55115	ERR10020314	Wairakei Terraces Feature 5	Wairakei-Tauhara	Dec 2013	8.5	89.0	143	2.2
P1.0011	PRJEB55115	ERR10020306	Wairakei Terraces Feature 13	Wairakei-Tauhara	Aug 2013	8.3	62.9	192	3.1
P1.0148	PRJEB24353	ERR2240256	Wairakei Terraces Feature 13	Wairakei-Tauhara	Dec 2013	8.5	65.5	226	3.7
P1.0010	PRJEB55115	ERR10020305	Wairakei Terraces Feature 14	Wairakei-Tauhara	Aug 2013	8.3	96.7	230	1.9
P1.0149	PRJEB24353	ERR2240257	Wairakei Terraces Feature 14	Wairakei-Tauhara	Dec 2013	8.5	95.2	119	2.6
P1.0019	PRJEB24353	ERR2240145	Radiata Pool	Ngatamariki	Aug 2013	7.5	63.3	247	3.0
P1.0482	PRJEB55115	ERR10020323	Radiata Pool	Ngatamariki	Jun 2014	7.7	60.0	306	3.5
P1.0054	PRJEB24353	ERR2240180	Champagne Pool	Waiotapu	Sep 2013	5.5	72.4	61	1.8
P1.0236	PRJEB55115	ERR10020321	Champagne Pool	Waiotapu	Feb 2014	5.5	76.4	53	1.6
P1.0472	PRJEB55115	ERR10020322	Champagne Pool	Waiotapu	May 2014	5.3	72.9	83	2.0
P1.0675	PRJEB55115	ERR10020324	Champagne Pool	Waiotapu	Sep 2014	5.3	74.5	48	1.0
P1.0923	PRJEB55115	ERR10020325	Champagne Pool	Waiotapu	Dec 2014	5.2	74.0	64	1.5

Table S2. Disturbed features and associated metadata at Waimangu geothermal field. This subset of samples were taken from Waimangu Stream and Inferno Crater Lake, before (August 2013) and during (October 2013) an overflow of hydrothermal fluid from Inferno Crater Lake into the stream. Accessions for sample sequences are also included.

Sample ID	Study accession	Run accession	Feature name	Geothermal field	Date	pH	Temp (°C)	OTU Richness	Shannon
P1.0027	PRJEB24353	ERR2240153	Waimangu Stream Feature 2	Waimangu	Aug 2013	5.9	49.1	178	2.7
P1.0110	PRJEB55115	ERR10020313	Waimangu Stream Feature 2	Waimangu	Oct 2013	6.1	48.8	191	2.4
P1.0028	PRJEB24353	ERR2240154	Waimangu Stream Feature 3	Waimangu	Aug 2013	6.5	49.8	176	2.6
P1.0109	PRJEB55115	ERR10020312	Waimangu Stream Feature 3	Waimangu	Oct 2013	6.7	48.5	221	2.4
P1.0037	PRJEB24353	ERR2240163	Inferno Crater Lake	Waimangu	Aug 2013	2.4	41.0	51	1.8
P1.0108	PRJEB55115	ERR10020311	Inferno Crater Lake	Waimangu	Oct 2013	2.6	69.1	42	1.6
P1.0029	PRJEB24353	ERR2240155	Waimangu Stream Feature 4	Waimangu	Aug 2013	6.7	49.9	221	2.8
P1.0107	PRJEB55115	ERR10020310	Waimangu Stream Feature 4	Waimangu	Oct 2013	3.0	56.6	324	3.4
P1.0030	PRJEB24353	ERR2240156	Waimangu Stream Feature 5	Waimangu	Aug 2013	7.4	49.6	224	3.0
P1.0106	PRJEB55115	ERR10020309	Waimangu Stream Feature 5	Waimangu	Oct 2013	3.0	55.2	248	2.8
P1.0032	PRJEB24353	ERR2240158	Waimangu Stream Feature 6	Waimangu	Aug 2013	7.4	37.0	1049	3.9
P1.0105	PRJEB55115	ERR10020308	Waimangu Stream Feature 6	Waimangu	Oct 2013	6.5	39.5	1191	4.8
P1.0031	PRJEB24353	ERR2240157	Waimangu Stream Feature 7	Waimangu	Aug 2013	6.9	33.7	848	3.8
P1.0104	PRJEB55115	ERR10020307	Waimangu Stream Feature 7	Waimangu	Oct 2013	6.6	34.2	834	4.5

Table S3. Disturbed features and associated metadata at Waikite geothermal field. These features were analysed before and after a long-term, anthropogenic disturbance was introduced to a geothermal wetland undergoing rehabilitation. Accessions for sample sequences are also included.

Sample ID	Study accession	Run accession	Feature name	Description	Geothermal field	Date	pH	Temp (°C)	OTU richness	Shannon
P1.0130	PRJEB24353	ERR2240240	Waikite Restoration Feature 3	Stream upgradient	Waikite	Dec 2013	8.6	44.3	766	4.5
P1.0171	PRJEB55115	ERR10020316	Waikite Restoration Feature 3	Stream upgradient	Waikite	Jan 2014	8.4	39.9	906	4.2
P1.1004	PRJEB55115	ERR10020329	Waikite Restoration Feature 3	Stream upgradient	Waikite	Apr 2015	8.5	37.8	1358	5.3
P1.1017	PRJEB55115	ERR10020337	Waikite Restoration Feature 3	Stream upgradient	Waikite	Oct 2016	8.3	39.5	805	4.1
P1.0131	PRJEB24353	ERR2240241	Waikite Restoration Feature 4	Wetland - inlet	Waikite	Dec 2013	8.5	47.2	1118	4.8
P1.0172	PRJEB55115	ERR10020317	Waikite Restoration Feature 4	Wetland - inlet	Waikite	Jan 2014	8.4	42.2	1308	5.0
P1.1005	PRJEB55115	ERR10020330	Waikite Restoration Feature 4	Wetland - inlet	Waikite	Apr 2015	8.3	39.0	1709	5.6
P1.1016	PRJEB55115	ERR10020336	Waikite Restoration Feature 4	Wetland - inlet	Waikite	Oct 2016	8.1	41.3	1030	4.2
P1.0132	PRJEB24353	ERR2240242	Waikite Restoration Feature 5	Wetland - hot	Waikite	Dec 2013	8.4	70.0	999	5.1
P1.0173	PRJEB55115	ERR10020318	Waikite Restoration Feature 5	Wetland - hot	Waikite	Jan 2014	8.4	67.9	826	5.1
P1.1007	PRJEB55115	ERR10020332	Waikite Restoration Feature 5	Wetland - hot	Waikite	Apr 2015	8.2	65.7	887	4.8
P1.1015	PRJEB55115	ERR10020335	Waikite Restoration Feature 5	Wetland - hot	Waikite	Oct 2016	8.1	65.5	667	4.4
P1.0133	PRJEB24353	ERR2240243	Waikite Restoration Feature 6	Wetland - warm	Waikite	Dec 2013	8.3	53.2	762	4.4
P1.0174	PRJEB55115	ERR10020319	Waikite Restoration Feature 6	Wetland - warm	Waikite	Jan 2014	8.3	43.8	1606	5.9
P1.1002	PRJEB55115	ERR10020327	Waikite Restoration Feature 6	Wetland - warm	Waikite	Apr 2015	7.7	23.3	2613	6.2
P1.1014	PRJEB55115	ERR10020334	Waikite Restoration Feature 6	Wetland - warm	Waikite	Oct 2016	7.5	29.9	1138	5.2
P1.0135	PRJEB24353	ERR2240245	Waikite Restoration Feature 8	Wetland - outlet	Waikite	Dec 2013	8.6	46.0	1375	5.5
P1.0176	PRJEB55115	ERR10020320	Waikite Restoration Feature 8	Wetland - outlet	Waikite	Jan 2014	8.6	42.8	1509	5.6
P1.1001	PRJEB55115	ERR10020326	Waikite Restoration Feature 8	Wetland - outlet	Waikite	Apr 2015	7.7	27.5	2877	7.0
P1.1013	PRJEB55115	ERR10020333	Waikite Restoration Feature 8	Wetland - outlet	Waikite	Oct 2016	7.6	26.0	1242	4.4
P1.0129	PRJEB24353	ERR2240239	Waikite Restoration Feature 2	Pig Scorcher	Waikite	Dec 2013	7.2	95.8	189	2.7
P1.0170	PRJEB55115	ERR10020315	Waikite Restoration Feature 2	Pig Scorcher	Waikite	Jan 2014	7.2	93.8	277	2.4
P1.1003	PRJEB55115	ERR10020328	Waikite Restoration Feature 2	Pig Scorcher	Waikite	Apr 2015	7.1	87.2	135	0.9
P1.0181	PRJEB24353	ERR2240282	Waikite Restoration Feature 13	Big Spring	Waikite	Jan 2014	7.5	96.5	284	3.9
P1.1006	PRJEB55115	ERR10020331	Waikite Restoration Feature 13	Big Spring	Waikite	Apr 2015	7.4	94.5	411	4.0

Table S4. pH features and associated metadata. Samples were taken from 12 geothermal features across the pH range commonly found in features from the Taupō Volcanic Zone (TVZ), Aotearoa-New Zealand (pH groups 3, 5, 7, and 9). All features had a similar temperature range of ~60-70 °C. Accessions for sample sequences are also included.

Sample ID	Study accession	Run accession	Feature name	pH group	Geothermal field	Date	pH	Temp (°C)	Richness	Shannon
P2.0019	PRJEB55115	ERR10020352	Whakarewarewa Feature 53	3	Rotorua	Feb 2016	3.0	60.1	39	1.1
P2.0024	PRJEB55115	ERR10020355	Whakarewarewa Feature 53	3	Rotorua	Apr 2016	3.1	59.1	41	2.2
P2.0036	PRJEB55115	ERR10020367	Whakarewarewa Feature 53	3	Rotorua	Jun 2016	3.1	57.2	37	1.6
P2.0048	PRJEB55115	ERR10020379	Whakarewarewa Feature 53	3	Rotorua	Aug 2016	3.0	50.8	30	1.8
P2.0060	PRJEB55115	ERR10020391	Whakarewarewa Feature 53	3	Rotorua	Oct 2016	2.9	53.0	28	1.6
P2.0017	PRJEB55115	ERR10020351	Whakarewarewa Feature 51	3	Rotorua	Feb 2016	3.3	70.3	24	1.3
P2.0026	PRJEB55115	ERR10020357	Whakarewarewa Feature 51	3	Rotorua	Apr 2016	3.6	67.1	25	1.6
P2.0038	PRJEB55115	ERR10020369	Whakarewarewa Feature 51	3	Rotorua	Jun 2016	4.0	66.3	27	1.7
P2.0050	PRJEB55115	ERR10020381	Whakarewarewa Feature 51	3	Rotorua	Aug 2016	4.6	60.0	45	2.1
P2.0062	PRJEB55115	ERR10020393	Whakarewarewa Feature 51	3	Rotorua	Oct 2016	4.8	60.6	39	1.5
P2.0023	PRJEB55115	ERR10020354	Te Puia Feature 11	3	Rotorua	Apr 2016	2.4	67.4	21	1.3
P2.0035	PRJEB55115	ERR10020366	Te Puia Feature 11	3	Rotorua	Jun 2016	2.5	66.1	23	1.2
P2.0047	PRJEB55115	ERR10020378	Te Puia Feature 11	3	Rotorua	Aug 2016	2.6	61.8	16	1.0
P2.0059	PRJEB55115	ERR10020390	Te Puia Feature 11	3	Rotorua	Oct 2016	2.5	64.0	19	0.9
P2.0002	PRJEB55115	ERR10020339	Waiotapu No.16 Feature 1	5	Waiotapu	Dec 2015	4.6	64.9	33	0.9
P2.0012	PRJEB55115	ERR10020347	Waiotapu No.16 Feature 1	5	Waiotapu	Feb 2016	4.6	67.5	36	0.9
P2.0032	PRJEB55115	ERR10020363	Waiotapu No.16 Feature 1	5	Waiotapu	Apr 2016	5.0	67.2	40	1.3
P2.0044	PRJEB55115	ERR10020375	Waiotapu No.16 Feature 1	5	Waiotapu	Jun 2016	4.9	67.0	34	1.1
P2.0056	PRJEB55115	ERR10020387	Waiotapu No.16 Feature 1	5	Waiotapu	Aug 2016	4.7	65.6	32	1.1
P2.0068	PRJEB55115	ERR10020399	Waiotapu No.16 Feature 1	5	Waiotapu	Oct 2016	4.7	68.0	35	1.2
P2.0003	PRJEB55115	ERR10020340	Artist's Palette Feature 1	5	Waiotapu	Dec 2015	5.1	59.4	90	1.4
P2.0011	PRJEB55115	ERR10020346	Artist's Palette Feature 1	5	Waiotapu	Feb 2016	4.8	67.9	48	1.0
P2.0033	PRJEB55115	ERR10020364	Artist's Palette Feature 1	5	Waiotapu	Apr 2016	4.9	66.5	63	1.3
P2.0045	PRJEB55115	ERR10020376	Artist's Palette Feature 1	5	Waiotapu	Jun 2016	4.8	64.7	87	1.7
P2.0057	PRJEB55115	ERR10020388	Artist's Palette Feature 1	5	Waiotapu	Aug 2016	4.7	66.9	74	1.4

Sample ID	Study accession	Run accession	Feature name	pH group	Geothermal field	Date	pH	Temp (°C)	Richness	Shannon
P2.0069	PRJEB55115	ERR10020400	Artist's Palette Feature 1	5	Waiotapu	Oct 2016	4.8	71.9	57	1.6
P2.0006	PRJEB55115	ERR10020342	Kuirau Park Feature 60	5	Rotorua	Dec 2015	5.2	59.2	70	1.9
P2.0015	PRJEB55115	ERR10020350	Kuirau Park Feature 60	5	Rotorua	Feb 2016	5.2	61.0	73	1.8
P2.0031	PRJEB55115	ERR10020362	Kuirau Park Feature 60	5	Rotorua	Apr 2016	5.3	56.6	90	2.0
P2.0043	PRJEB55115	ERR10020374	Kuirau Park Feature 60	5	Rotorua	Jun 2016	5.0	51.7	138	2.2
P2.0055	PRJEB55115	ERR10020386	Kuirau Park Feature 60	5	Rotorua	Aug 2016	4.9	45.8	119	1.8
P2.0067	PRJEB55115	ERR10020398	Kuirau Park Feature 60	5	Rotorua	Oct 2016	4.5	51.3	88	1.8
P2.0001	PRJEB55115	ERR10020338	Whangapoa Feature 1	7	Atiamuri	Dec 2015	7.6	63.3	123	2.5
P2.0010	PRJEB55115	ERR10020345	Whangapoa Feature 1	7	Atiamuri	Feb 2016	7.3	65.3	114	2.2
P2.0028	PRJEB55115	ERR10020359	Whangapoa Feature 1	7	Atiamuri	Apr 2016	7.1	63.8	90	2.0
P2.0040	PRJEB55115	ERR10020371	Whangapoa Feature 1	7	Atiamuri	Jun 2016	7.1	61.0	129	2.8
P2.0052	PRJEB55115	ERR10020383	Whangapoa Feature 1	7	Atiamuri	Aug 2016	7.1	60.8	147	3.0
P2.0064	PRJEB55115	ERR10020395	Whangapoa Feature 1	7	Atiamuri	Oct 2016	7.6	62.7	122	2.7
P2.0013	PRJEB55115	ERR10020348	Kuirau Park Feature 87	7	Rotorua	Feb 2016	6.6	63.8	196	2.3
P2.0029	PRJEB55115	ERR10020360	Kuirau Park Feature 87	7	Rotorua	Apr 2016	6.9	61.4	171	1.4
P2.0041	PRJEB55115	ERR10020372	Kuirau Park Feature 87	7	Rotorua	Jun 2016	7.1	57.5	380	3.3
P2.0053	PRJEB55115	ERR10020384	Kuirau Park Feature 87	7	Rotorua	Aug 2016	6.6	62.0	306	2.4
P2.0065	PRJEB55115	ERR10020396	Kuirau Park Feature 87	7	Rotorua	Oct 2016	6.8	58.5	329	2.5
P2.0005	PRJEB55115	ERR10020341	Kuirau Park Feature 101	7	Rotorua	Dec 2015	7.1	70.6	101	1.4
P2.0014	PRJEB55115	ERR10020349	Kuirau Park Feature 101	7	Rotorua	Feb 2016	7.3	72.4	64	1.5
P2.0030	PRJEB55115	ERR10020361	Kuirau Park Feature 101	7	Rotorua	Apr 2016	7.1	71.4	138	1.8
P2.0042	PRJEB55115	ERR10020373	Kuirau Park Feature 101	7	Rotorua	Jun 2016	7.3	68.7	141	2.8
P2.0054	PRJEB55115	ERR10020385	Kuirau Park Feature 101	7	Rotorua	Aug 2016	7.0	67.9	151	2.8
P2.0066	PRJEB55115	ERR10020397	Kuirau Park Feature 101	7	Rotorua	Oct 2016	7.2	68.3	133	2.2
P2.0007	PRJEB55115	ERR10020343	Whakarewarewa Feature 45	9	Rotorua	Dec 2015	8.9	69.5	219	3.2
P2.0025	PRJEB55115	ERR10020356	Whakarewarewa Feature 45	9	Rotorua	Apr 2016	9.0	66.2	175	3.1
P2.0037	PRJEB55115	ERR10020368	Whakarewarewa Feature 45	9	Rotorua	Jun 2016	8.7	64.7	201	3.1
P2.0049	PRJEB55115	ERR10020380	Whakarewarewa Feature 45	9	Rotorua	Aug 2016	8.7	64.5	232	3.4
P2.0061	PRJEB55115	ERR10020392	Whakarewarewa Feature 45	9	Rotorua	Oct 2016	8.8	65.5	254	3.2

Sample ID	Study accession	Run accession	Feature name	pH group	Geothermal field	Date	pH	Temp (°C)	Richness	Shannon
P2.0009	PRJEB55115	ERR10020344	Whakarewarewa Feature 1	9	Rotorua	Dec 2015	8.3	63.4	101	1.9
P2.0027	PRJEB55115	ERR10020358	Whakarewarewa Feature 1	9	Rotorua	Apr 2016	8.4	61.1	168	2.9
P2.0039	PRJEB55115	ERR10020370	Whakarewarewa Feature 1	9	Rotorua	Jun 2016	8.0	70.3	108	2.5
P2.0051	PRJEB55115	ERR10020382	Whakarewarewa Feature 1	9	Rotorua	Aug 2016	7.8	78.1	105	2.5
P2.0063	PRJEB55115	ERR10020394	Whakarewarewa Feature 1	9	Rotorua	Oct 2016	8.4	80.6	102	2.3
P2.0022	PRJEB55115	ERR10020353	Ohaaki Feature 2	9	Ohaaki	Apr 2016	9.0	70.3	31	0.9
P2.0034	PRJEB55115	ERR10020365	Ohaaki Feature 2	9	Ohaaki	Jun 2016	8.9	66.5	48	1.1
P2.0046	PRJEB55115	ERR10020377	Ohaaki Feature 2	9	Ohaaki	Aug 2016	8.4	65.0	43	1.1
P2.0058	PRJEB55115	ERR10020389	Ohaaki Feature 2	9	Ohaaki	Oct 2016	9.0	67.9	77	1.7

Table S5. Variation in physicochemistry, alpha diversity and taxon relative abundance of control geothermal features. Variation is presented as standard deviation (SD) from the mean of values measured for pH, temperature (°C), OTU richness, Shannon diversity index, and dominant taxa (>1 % average relative abundance across all samples in this category). Alpha diversity and relative abundances of taxa were calculated via amplicon sequencing of the 16S rRNA gene from DNA extracted from these ecosystems.

Geothermal feature	Wairakei Feature 5		Wairakei Feature 13		Wairakei Feature 14		Radiata Pool		Champagne Pool	
	Mean	SD	Mean	SD	Mean	SD	Mean	SD	Mean	SD
pH	8.3	0.2	8.4	0.2	8.4	0.2	7.6	0.2	5.3	0.1
Temperature (°C)	91.0	2.8	64.2	1.8	96.0	1.1	61.7	2.3	74.0	1.6
OTU richness	106.5	51.6	209.0	24.0	174.5	78.5	276.5	41.7	61.8	13.4
Shannon index	1.7	0.7	3.4	0.4	2.2	0.5	3.3	0.3	1.6	0.4
Aquificota (%)	96.1	2.8	10.3	3.9	71.8	3.8	60.0	8.0	95.9	2.5
Armatimonadota (%)	0.0	0.0	7.5	7.5	0.2	0.1	0.3	0.0	0.0	0.0
Bacteroidota (%)	0.7	1.0	18.5	4.3	13.1	16.7	1.4	0.8	0.0	0.0
Chlorobiota (%)	0.0	0.0	8.2	0.4	0.0	0.0	5.9	2.1	0.0	0.0
Chloroflexota (%)	0.0	0.1	16.3	5.1	0.2	0.2	0.8	0.1	0.0	0.0
Deinococcota (%)	0.5	0.5	20.9	5.6	7.7	9.9	1.4	0.2	0.0	0.0
Pseudomonadota (%)	1.3	1.2	7.8	2.4	4.4	2.0	5.0	0.5	0.2	0.1
Thermodesulfobacteriota (%)	0.0	0.0	0.0	0.0	0.1	0.1	5.2	0.8	1.9	1.6

Table S6. Variation in physicochemistry, alpha diversity and taxon relative abundance of Waimangu geothermal features. Variation is presented as standard deviation (SD) from the mean of values measured for pH, temperature (°C), OTU richness, Shannon diversity index, and dominant taxa (>1 % average relative abundance across all samples in this category). Alpha diversity and relative abundances of taxa were calculated via amplicon sequencing of the 16S rRNA gene from DNA extracted from these ecosystems.

Geothermal feature	Waimangu Feature 2		Waimangu Feature 3		Inferno Crater Lake		Waimangu Feature 4		Waimangu Feature 5		Waimangu Feature 6		Waimangu Feature 7	
	Mean	SD	Mean	SD	Mean	SD	Mean	SD	Mean	SD	Mean	SD	Mean	SD
pH	6.0	0.1	6.6	0.1	2.5	0.1	4.9	2.6	5.2	3.1	6.9	0.6	6.8	0.2
Temperature (°C)	49.0	0.2	49.2	0.9	55.1	19.9	53.3	4.7	52.4	4.0	38.3	1.8	34.0	0.4
OTU richness	184.5	9.2	198.5	31.8	46.5	6.4	272.5	72.8	236.0	17.0	1120.0	100.4	841.0	9.9
Shannon index	2.5	0.2	2.5	0.2	1.7	0.1	3.1	0.4	2.9	0.2	4.4	0.7	4.1	0.5
Aquificota (%)	23.2	3.1	24.9	5.5	4.0	5.3	27.0	2.4	27.8	7.5	16.8	12.3	11.8	13.9
Bacteroidota (%)	1.9	0.1	2.2	0.4	0.1	0.1	5.6	2.5	2.9	0.2	9.9	7.2	18.6	14.2
Chlorobiota (%)	1.4	0.1	2.0	1.6	0.0	0.0	5.5	5.6	3.2	2.8	1.9	0.8	1.2	1.3
Cyanobacteria (%)	7.4	9.1	10.2	11.0	0.0	0.0	14.2	14.1	9.2	11.7	4.4	3.6	3.7	4.4
Euryarchaeota (%)	0.0	0.0	0.0	0.0	8.6	2.7	0.4	0.5	0.0	0.0	0.0	0.0	0.0	0.0
Parcubacteria (%)	0.7	0.6	0.8	0.3	0.0	0.0	1.3	1.5	1.0	0.9	2.4	1.4	0.8	0.3
Pseudomonadota (%)	63.2	11.1	57.8	13.6	36.9	51.9	35.7	4.6	52.4	13.3	52.6	7.0	56.1	3.4
Thermoproteota (%)	0.0	0.0	0.0	0.0	46.1	55.9	6.3	8.8	0.6	0.8	0.1	0.2	0.0	0.1

Table S7. Variation in physicochemistry, alpha diversity and taxon relative abundance of Waikite geothermal features. Variation is presented as standard deviation (SD) from the mean of values measured for pH, temperature (°C), OTU richness, Shannon diversity index, and dominant taxa (>1 % average relative abundance across all samples in this category). Alpha diversity and relative abundances of taxa were calculated via amplicon sequencing of the 16S rRNA gene from DNA extracted from these ecosystems.

Geothermal feature	Waikite Feature 3		Waikite Feature 4		Waikite Feature 5		Waikite Feature 6		Waikite Feature 8		Pig Scorcher		Big Spring	
	Mean	SD	Mean	SD	Mean	SD	Mean	SD	Mean	SD	Mean	SD	Mean	SD
pH	8.4	0.1	8.3	0.2	8.3	0.2	7.9	0.4	8.1	0.6	7.2	0.1	7.5	0.0
Temperature (°C)	40.4	2.8	42.4	3.5	67.3	2.1	37.6	13.5	35.6	10.3	92.3	4.5	95.5	1.4
OTU richness	958.8	272.6	1291.3	301.7	844.8	138.5	1529.8	800.4	1750.8	758.7	200.3	71.7	347.5	89.8
Shannon index	4.5	0.6	4.9	0.6	4.8	0.3	5.5	0.8	5.6	1.1	2.0	1.0	3.9	0.0
Aquificota (%)	0.1	0.0	0.0	0.0	1.4	0.5	0.0	0.0	0.0	0.0	76.5	19.3	45.6	1.8
Bacillota (%)	0.7	0.7	0.8	0.7	4.2	3.9	0.8	0.8	1.1	0.8	0.4	0.3	5.8	6.4
Bacteria (%)	0.7	0.5	0.9	0.3	1.7	0.3	3.2	2.3	1.5	1.3	2.0	3.2	0.6	0.0
Bacteroidota (%)	10.4	2.7	10.2	2.7	22.0	5.8	17.0	15.9	14.7	7.3	0.7	0.5	4.9	2.8
Chlamydiota (%)	0.4	0.3	0.6	0.4	1.5	2.8	1.9	3.0	2.5	3.8	0.0	0.0	0.3	0.4
Chlorobiota (%)	0.7	0.4	0.8	0.5	5.2	6.7	2.4	2.1	1.0	0.9	0.2	0.0	0.5	0.4
Chloroflexota (%)	0.5	0.3	0.5	0.3	5.9	5.8	1.4	0.9	0.4	0.3	0.3	0.1	1.1	0.6
Cyanobacteria (%)	10.0	7.6	5.3	4.5	2.6	4.5	6.9	7.9	2.5	1.7	0.9	1.1	0.6	0.1
Deinococcota (%)	0.1	0.1	0.2	0.1	3.4	3.3	0.1	0.1	0.1	0.1	12.0	16.1	8.1	3.5
Parcubacteria (%)	1.4	1.0	2.3	1.8	6.0	1.9	9.2	9.9	8.0	9.4	0.2	0.1	1.5	1.0
Planctomycetota (%)	1.3	0.7	1.6	0.4	0.5	0.3	1.2	1.0	1.6	0.8	0.0	0.0	0.7	0.2
Pseudomonadota (%)	65.9	10.5	68.0	10.9	27.6	7.0	39.6	20.4	52.8	12.4	3.2	2.4	22.5	9.8
Verrucomicrobiota (%)	2.3	1.4	2.9	1.2	1.6	0.9	4.8	4.7	2.8	0.7	0.0	0.0	1.1	0.5
Woesearchaeota (%)	0.1	0.1	0.5	0.4	1.8	1.4	2.3	2.2	1.9	1.6	0.0	0.0	0.1	0.2

Table S8. Variation in physicochemistry, alpha diversity and taxon relative abundance of geothermal features across the pH range. Variation is presented as standard deviation (SD) from the mean of values measured for pH, temperature (°C), OTU richness, Shannon diversity index, and dominant taxa (>1 % average relative abundance across all samples in this category). Alpha diversity and relative abundances of taxa were calculated via amplicon sequencing of the 16S rRNA gene from DNA extracted from these ecosystems.

pH group	3		3		3		5		5		5	
Geothermal feature	Whakarewarewa Feature 53		Whakarewarewa Feature 51		Te Puia Feature 11		Waiotapu No.16 Feature 1		Artist's Palette Feature 1		Kuirau Park Feature 60	
	Mean	SD	Mean	SD	Mean	SD	Mean	SD	Mean	SD	Mean	SD
pH	3.0	0.1	4.1	0.6	2.5	0.1	4.7	0.2	4.8	0.1	5.0	0.3
Temperature (°C)	56.0	4.0	64.9	4.4	64.8	2.5	66.7	1.2	66.2	4.1	54.3	5.7
OTU richness	35.0	5.7	32.0	9.4	19.8	3.0	35.0	2.8	69.8	16.8	96.3	26.8
Shannon index	1.7	0.4	1.6	0.3	1.1	0.1	1.1	0.2	1.4	0.2	1.9	0.1
Aquificota (%)	21.1	33.1	24.1	20.2	0.6	0.5	86.1	6.3	80.8	11.1	46.6	24.7
Armatimonadota (%)	0.0	0.0	0.0	0.0	0.0	0.0	0.0	0.0	0.0	0.0	0.0	0.0
Bacteroidota (%)	0.0	0.0	0.0	0.0	0.0	0.0	0.0	0.0	0.1	0.1	0.1	0.1
Deinococcota (%)	0.0	0.0	0.0	0.0	0.0	0.0	0.0	0.0	0.0	0.0	0.0	0.0
Euryarchaeota (%)	11.0	7.5	22.5	12.6	5.1	5.0	3.8	2.3	3.7	1.8	1.1	0.8
Pseudomonadota (%)	59.5	27.9	0.4	0.4	0.0	0.0	0.0	0.1	2.0	2.7	49.0	24.4
Thermodesulfobacteriota (%)	0.0	0.0	0.0	0.0	0.0	0.0	0.0	0.0	0.1	0.1	0.1	0.2
Thermoproteota (%)	0.1	0.2	49.7	33.6	89.7	6.2	10.0	4.4	13.0	10.9	0.3	0.1
Thermotogota (%)	8.0	4.0	0.1	0.1	0.0	0.0	0.0	0.0	0.1	0.1	0.3	0.3

pH group	7		7		7		9		9		9	
Geothermal feature	Whangapoa Feature 1		Kuirau Park Feature 87		Kuirau Park Feature 101		Whakarewarewa Feature 45		Whakarewarewa Feature 1		Ohaaki Feature 2	
	Mean	SD	Mean	SD	Mean	SD	Mean	SD	Mean	SD	Mean	SD
pH	7.3	0.2	6.8	0.2	7.2	0.1	8.8	0.1	8.2	0.3	8.8	0.3
Temperature (°C)	62.8	1.7	60.6	2.6	69.9	1.8	66.1	2.0	70.7	8.6	67.4	2.3
OTU richness	120.8	18.7	276.4	89.4	121.3	32.8	216.2	30.1	116.8	28.8	49.8	19.5
Shannon index	2.5	0.4	2.4	0.7	2.1	0.6	3.2	0.1	2.4	0.4	1.2	0.4
Aquificota (%)	3.8	0.7	71.7	12.4	74.8	7.3	35.6	5.2	39.3	28.2	73.2	42.3
Armatimonadota (%)	28.0	10.4	0.1	0.0	0.2	0.1	0.0	0.0	0.0	0.0	1.0	0.6
Bacteroidota (%)	9.5	6.4	2.2	1.8	0.4	0.2	0.9	0.3	2.0	2.9	0.3	0.2
Deinococcota (%)	38.0	16.1	1.0	1.3	10.4	9.1	5.6	3.3	33.1	27.3	19.5	33.5
Euryarchaeota (%)	0.0	0.0	0.5	0.5	0.1	0.1	0.0	0.0	0.1	0.1	0.0	0.0
Pseudomonadota (%)	6.0	4.0	4.0	1.9	1.0	0.8	29.3	7.6	3.9	3.4	1.4	0.9
Thermodesulfobacteriota (%)	0.1	0.0	2.6	1.6	1.8	1.2	11.4	2.9	9.2	5.0	3.0	5.5
Thermoproteota (%)	0.0	0.0	0.4	0.3	7.7	5.4	0.1	0.0	0.3	0.1	0.0	0.0
Thermotogota (%)	0.4	0.1	0.4	0.2	0.5	0.3	5.8	1.8	2.8	1.3	0.3	0.4
